# Supplementary material for: Sunlight-driven simultaneous CO2 reduction and water oxidation using indium-organic framework heterostructures
Source: Nat Commun. 2025 Mar 16;16:2601. doi: 10.1038/s41467-025-57742-5 (PMC11911404; doi:10.1038/s41467-025-57742-5)
Supplement: Supplementary file 1 — Supplementary Information [file 41467_2025_57742_MOESM1_ESM.pdf]

Supplementary materials

Sunlight-driven simultaneous CO_2 reduction and water oxidation using indium-organic framework heterostructures

Zhongjie Cai[1], Hongwei Liu[2], Jiajun Dai[3], Bao Li[1], Liming Yang[1], Jingyu Wang[1,*], Huaiyong Zhu[4]

[1]Key Laboratory of Material Chemistry for Energy Conversion and Storage (Ministry of Education), Hubei Key Laboratory of Material Chemistry and Service Failure, School of Chemistry and Chemical Engineering, Huazhong University of Science and Technology, Wuhan 430074, China

[2]Australian Centre for Microscopy and Microanalysis University of Sydney, Chippendale, NSW 2006, Australia

[3]Institute of Chemistry and Biochemistry, Freie Universität Berlin, Arnimallee 22, Berlin 14195, Germany

[4]School of Chemistry and Physics, Queensland University of Technology, QLD 4001, Australia

*E-mail: wangjingyu@hust.edu.cn.

Table of contents

Supplementary Notes

Supplementary Note 1. Characterizations…………………………...3-5

Supplementary Note 2. DFT Calculation………………………………6

Supplementary Figs 1 to 61…………………………………………7-55

Supplementary Tables 1 to 11………………………………………56-66

Supplementary References………………………………………...67-69

Supplementary Note 1. Characterizations

The proton nuclear magnetic resonance (^1H NMR) spectra of the solution on a Bruker Avance III HD NMR spectrometer. Fourier transform infrared (FT-IR) spectra were collected using KBr pellets on a VERTEX 70 FT-IR spectrometer. A Rigaku Smart Lab-SE X-ray diffractometer with Cu Kα radiation (λ = 1.54056 Å) were recorded with the powder X-ray diffraction (PXRD) patterns of the samples. Thermal analysis was performed on a Pyris1 TGA thermal analyzer at a temperature range of 25 to 900 °C under a nitrogen atmosphere with a heating rate of 10 $°C \cdot min^{-1}$. The N_2 and CO_2 sorption isotherm were measured on a Micromeritics ASAP 2020 surface area and a pore size analyzer. Scanning electron microscopy (SEM) images were obtained by using a field emission scanning electron microscope (FEI Sirion 200, USA) at 5 kV. Surface chemical analysis was performed on an X-ray photoelectron spectrometer (Thermo VG scientific ESCA MultiLab-2000) and all the binding energies were calibrated by the C *1s* peak at 284.8 eV from adventitious carbon. The transmission electron microscopy (TEM) images of samples were recorded on a Tecnai G2 F30 microscope (FEI, Holland). Photoluminescence (PL) emission spectra were collected by a Hitachi F-7000 spectrofluorometer at the excitation wavelength of 365 and 515 nm. PL decays were collected from these sample suspensions on an Edinburgh FLS920 spectrometer, where the probe positioned at 650 nm emission under 375 nm laser excitation. UV-vis diffuse reflectance spectra (DRS) were obtained using a UV-vis spectrophotometer (UV-3600, Shimadzu, Japan). The electrochemical and photoelectrochemical properties of the sample were tested using an electrochemical workstation (CHI650E, Chenhua Com., China) with a standard three-electrode system. A Pt wire and Ag/AgCl (KCl solution, 3.5 M) were used as the counter and reference electrodes, respectively. 5 mg of a catalyst was dispersed into 1 mL of 1:1

isopropanol/H_2O containing 10 μL of Nafion. Then, 50 μL of the above suspension was coated on an ITO glass as a working electrode. Electrochemical impedance spectra (EIS) were obtained in 0.1 M KCl electrolyte containing 5 mM $Fe(CN)_6^{3-}/Fe(CN)_6^{4-}$. Photocurrent signals were detected in 1 M Na_2SO_4 solution during light-on and light-off cycles. The Mott-Schottky plots were obtained on an IM 6 electrochemical system in 0.2 M Na_2SO_4 (pH=6.8 ± 0.05) electrolyte at the frequencies of 500, 1000, and 1500 Hz. The energy levels versus normal hydrogen electrode (NHE) are calibrated by the difference of 0.2046 V between NHE and Ag/AgCl electrode. Photocurrent experiments were operated on a electrochemical station under a chopped light (300 W Xe lamp with a 400 nm cutoff filter, 0.30 W cm^{-2}) under open-circuit potentials. The Linear sweep voltammetry (LSV) curves were recorded in CO_2-saturated and Ar-saturated 0.1 M KOH solutions with the scan rate of 100 mV s^{-1} in the H-cell system.

Femtosecond transient absorption (TA) spectroscopy measurements were performed by a regenerative amplified Ti: sapphire laser system (Coherent; 800 nm, 35 fs, 6 mJ/pulse, and 1 kHz repetition rate), nonlinear frequency mixing techniques and the Helios spectrometer (Ultrafast Systems LLC) at room temperature. The samples were suspended in DMF. Briefly, the 800 nm output pulse from the regenerative amplifier was split into two parts with a 50% beam splitter. One part was directed to pump a TOPAS Optical Parametric Amplifier (OPA), which generates a wavelength-tunable laser pulse from 250 nm to 2.5 μm as pump beam. Here, a 380 nm laser was selected as the pump beam. The reflected 800 nm beam was split again into two parts. One part with less than 10% was attenuated with a neutral density filter and focused into a 2 mm thick CaF_2 window to generate a white light continuum (WLC) from 320 nm to 650 nm used for the probe beam. The probe beam was focused onto the sample using an Al parabolic reflector. Then, the probe beam was collimated and focused into

a fiber-coupled spectrometer with CMOS sensors and detected at a frequency of 1 kHz. The intensity of the pump pulse used in the experiment was controlled by a variable neutral-density filter wheel. The delay between the pump and probe pulses was controlled by a motorized delay stage. The pump pulses were chopped by a synchronized chopper at 500 Hz and the absorbance change was calculated with two adjacent probe pulses (pump-blocked and pump-unblocked).

In-situ DRIFTS spectra were recorded by a Tensor II FT-IR spectrometer (Thermo Fisher: Nicolet iS50, USA) in the range of 600-4000 cm^{-1}. Typically, the samples were dried overnight in vacuum at 373 K before testing, which removes any adsorbed impurities. After pre-treatment, the sample was placed into and sealed within the sample cell, which was purged with high-purity He gas to remove the air for 30 min. Subsequently, the mixture of CO_2 and H_2O vapor (bubbling of CO_2 gas through water) was introduced into the sample cell for 60 min, and the in-situ FTIR adsorption peaks spectra were recorded under dark condition. After that, the sample was illuminated under visible light (300 W Xe lamp with a 400 nm cutoff filter) with continuous introduction of CO_2 and H_2O vapor, and Time-resolved DRIFT spectra were recorded for 60 min.

In situ light-induced EPR spectra were measured by a Bruker-BioSpinE500 spectrometer at room temperature. (1) To amplify the EPR response caused by the photoelectrons of M68N, M68N@In-TCPP, and In-TCPP. Before the test, the power (20 mg) was introduced with CO_2 or N_2 flow for 30 min respectively, then sampled by capillaries and transferred into quartz tubes filled with CO_2 or N_2 correspondingly. The visible light irradiation was performed by a 300 W Xenon lamp. (2) To detect of possible radicals on the surface of M68N@In-TCPP or in the liquid during photocatalysis.

Supplementary Note 2. DFT Calculations

Our first-principles calculations are based on DFT with general gradient approximation (GGA) for exchange-correlation potential for which Perdew, Burke, and Ernzerhof (PBE)[1] exchange-correlation Functional using plane-wave basis VASP code[2]. The projector augmented wave (PAW) method is employed to describe the electron-ion interactions. A kinetic energy cutoff of 500 eV and Monkhorst-Pack k-point grids of 1×1×1 were used for the optimization. Meanwhile, the convergence thresholds were set to 10^{-5} eV and 10^{-2} eV/Å for the input energy and the force, respectively. The adsorption energy (E_{ads}) was calculated by $E_{ads} = E_{total} - E_{MOF/TCPP} - E_{molecule}$, where E_{total}, $E_{MOF/TCPP}$ and $E_{molecule}$ are the optimized energies of the adsorption structure, MOF/TCPP and molecule (CO or HCOOH) respectively. For the Gibbs free energy calculations, the entropic corrections and zero-point energy (ZPE) have been included. The Gibbs free energy is calculated using the following equation:

$$G = E_{DFT} + E_{ZPE} - TS \quad (1)$$

where G is the Gibbs free energy, E_{DFT} is the DFT-calculated total energy, E_{ZPE} is the zero point energy, S is the entropy, and T is room temperature (298.15 K).

Supplementary Figures

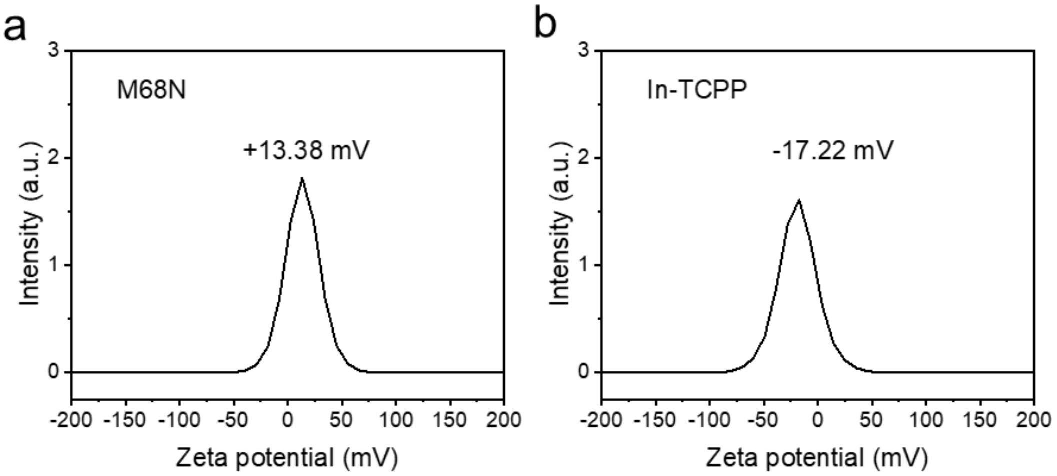

Supplementary Fig. 1. Zeta potential. Zeta potential measurements of NH_2-MIL-68(M68N) **a** In-TCPP **b** in EtOH solution. Source data are provided as a Source Data file.

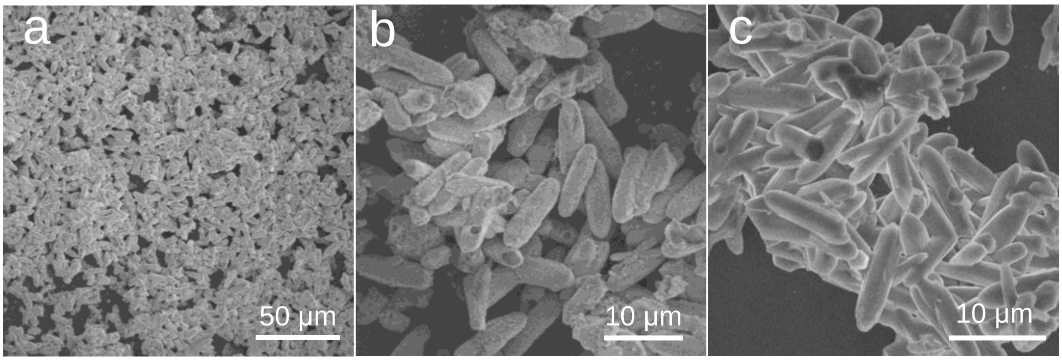

Supplementary Fig. 2. SEM. SEM images of **a-b** M68N after reaction in DMF solution for 5 h and **c** initial nucleation of M68N at 0.5 h.

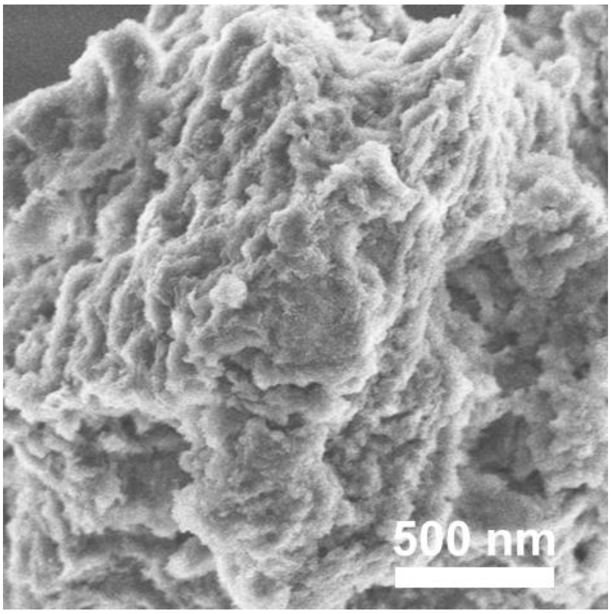

Supplementary Fig. 3. SEM. SEM images of In-TCPP after reaction in dimethylformamide (DMF) solution for 5 h.

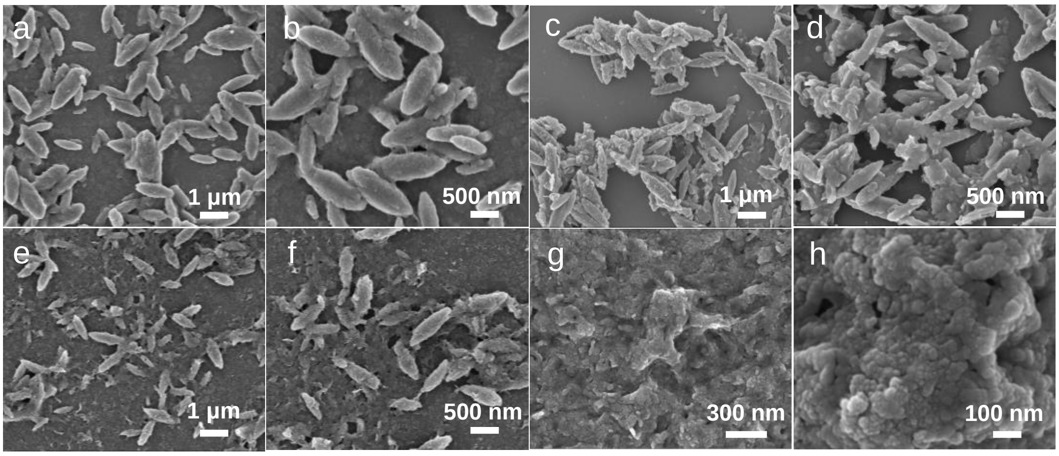

Supplementary Fig. 4. **SEM.** SEM images of M68N@InTCPP with various molar percentages of TCPP in the total amount of linkers (TCPP and NH_2-BDC). **a-b** 5 %; **c-d** 10 %; **e-f** 20 %; **g-h** 30 %. Note: the scare bar of g and h was 300 and 100 nm due to the In-TCPP morphology only being seen clearly in higher magnification than M68N.

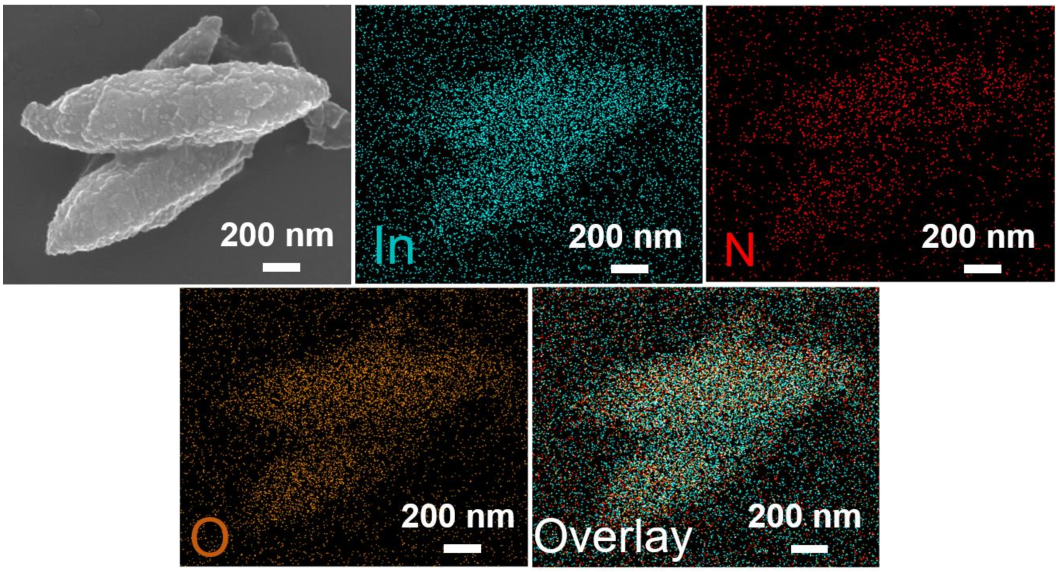

Supplementary Fig. 5. SEM. SEM images of M68N@In-TCPP and the corresponding element mapping distributions.

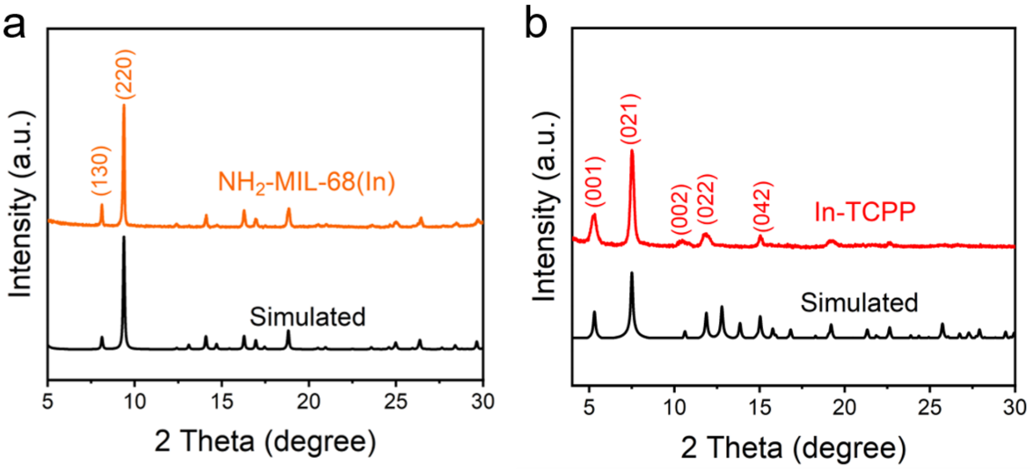

Supplementary Fig. 6. PXRD. The PXRD patterns of **a** M68N and **b** In-TCPP along with their simulated data. Source data are provided as a Source Data file.

The diffraction peaks at 8.2, and 9.5° are assigned to the (130), and (220) crystal planes of M68N, respectively. The diffraction peaks located at 5.3, 7.5, 11.8, and 15.0° are assigned to the (001), (021), (022), and (042) facets of In-TCPP, respectively. Source data are provided as a Source Data file.

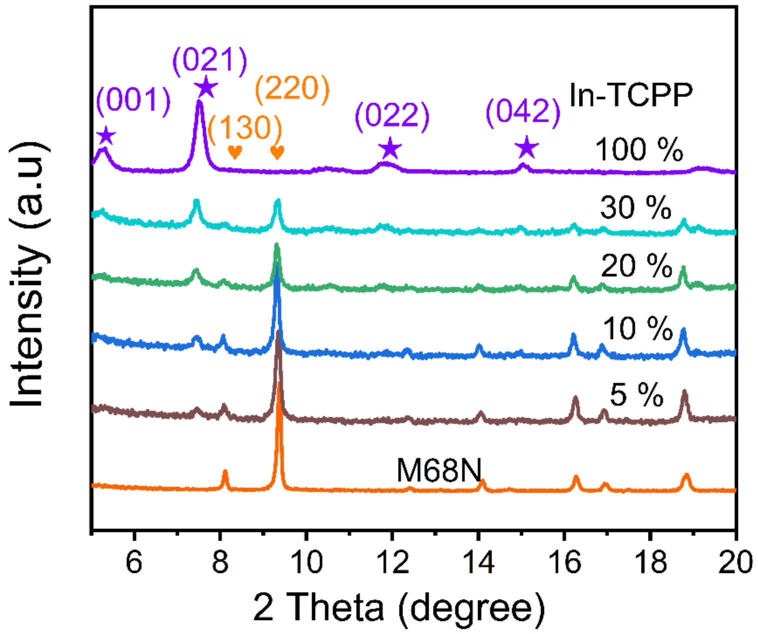

Supplementary Fig. 7. PXRD. PXRD patterns of M68N@InTCPP with various molar percentages of TCPP in the total amount of linkers (TCPP and NH_2-BDC). The diffraction intensity of (001) and (021) facets of In-TCPP increased with the molar percentage of the TCPP ligand. Source data are provided as a Source Data file.

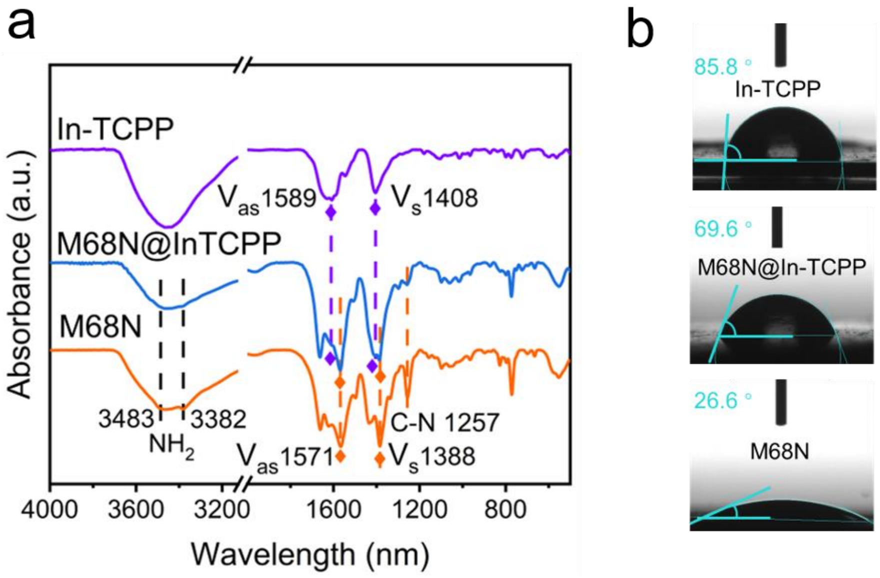

Supplementary Fig. 8. FT-IR and water contact angles. Chemical structure and surface wettability of M68N, In-TCPP, and M68N@In-TCPP. **a** FT-IR spectra. **b** Water contact angles measurement. Source data are provided as a Source Data file.

The Fourier transform infrared (FT-IR) spectrum of M68N shows characteristic peaks at 1571 and 1388 cm^{-1}, corresponding to the asymmetric (V_{as}) and symmetric (V_s) stretching vibration of coordinated carboxylic groups. The Fourier transform infrared (FT-IR) spectrum of M68N shows characteristic peaks at 1571 and 1388 cm^{-1}, corresponding to the asymmetric (V_{as}) and symmetric (V_s) stretching vibration of coordinated carboxylic groups (Supplementary Fig. 8a)[3]. Similar vibrational bands are observed in the FT-IR spectrum of In-TCPP. Surface wettability measurements, shown in Supplementary Fig. 8b, further support the presence of the In-TCPP shell in the heterostructure. M68N displays a significantly lower water contact angle (26.6°) than In-TCPP (85.8°) due to the hydrophilicity of -NH_2 groups. The water contact angle of the M68N@In-TCPP heterostructure is 69.8°, aligning closer to that of In-TCPP and confirming that the In-TCPP layer effectively covers the In-TCPP layer on the surface of the heterostructure.

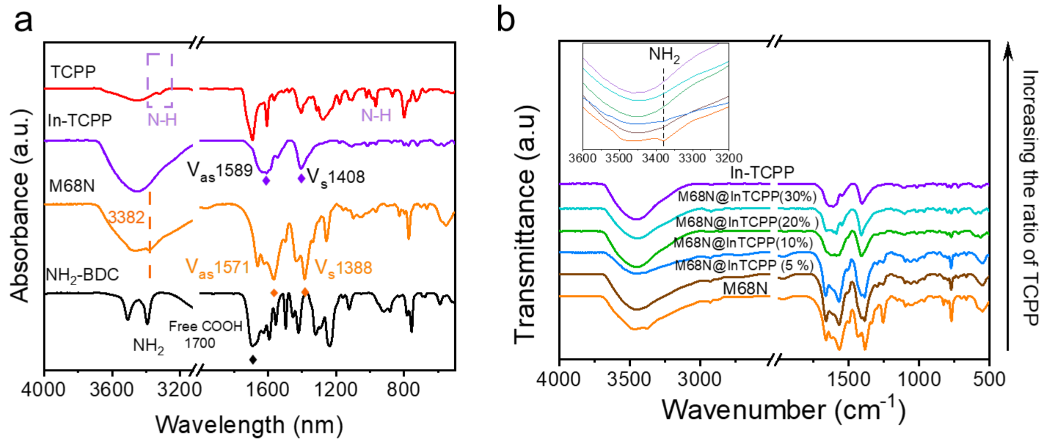

Supplementary Fig. 9. FT-IR. a FIIR spectra of TCPP, In-TCPP, M68N, and M68N@In-TCPP. **b** FIIR spectra of M68N@In-TCPP with various ratios of NH_2-BDC and TCPP ligands. Source data are provided as a Source Data file.

The absence of the free -COOH group signal at 1700 cm^{-1} in M68N or In-TCPP, present in NH_2-BDC or TCPP linkers, confirms successful MOF formation (Supplementary Fig. 9a)[4,5]. The symmetric and antisymmetric stretching vibrations of the -NH_2 group of NH_2-BDC at 3382 and 3483 cm^{-1} are retained in M68N@In-TCPP. The stretching vibration and bending vibration of N-H in the pyrrole of TCPP at 3321 and 964 cm^{-1} nearly disappear in In-TCPP (Supplementary Fig. 9a), confirming the coordination of N-H to indium at the centre of the porphyrin ring[6,7]. The signals of M68N in the M68N@In-TCPP gradually decrease with increasing TCPP linker ratio (Supplementary Fig. 9b). No additional absorption bands appear in the FT-IR spectra of M68N@In-TCPP (Supplementary Fig. 8a and Supplementary Fig. 9), indicating successful synthesis of the heterostructure without the formation of new chemical bonds between two linkers.

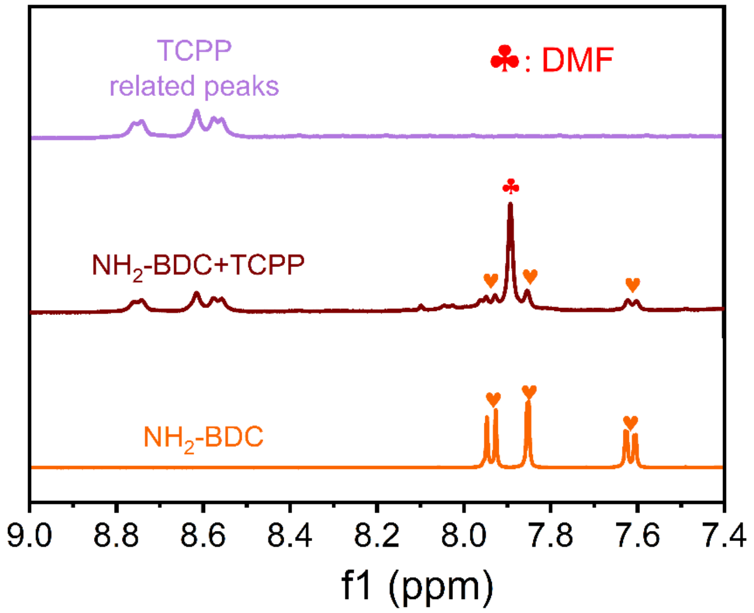

Supplementary Fig. 10. ^{1}HNMR. ^{1}HNMR spectra of NH_2-BDC and TCPP linker. NH_2-BDC+TCPP represents the two linkers experienced in the same synthetic procedure of M68N@In-TCPP hybrid. That is, the two linkers (NH_2-BDC and TCPP) were added to the reaction system in the absence of In, in order to study whether there is chemical reaction between the functional group of linkers. The signal at 7.9 ppm comes from the dimethylformamide (DMF) solvent in the reaction system. Source data are provided as a Source Data file.

^{1}H nuclear magnetic resonance (^{1}H NMR) spectra confirm the presence of characteristic peaks for NH_2-BDC and TCPP without additional signals or chemical shifts, supporting that functional groups only connect with the In node to form the heterostructure MOFs.

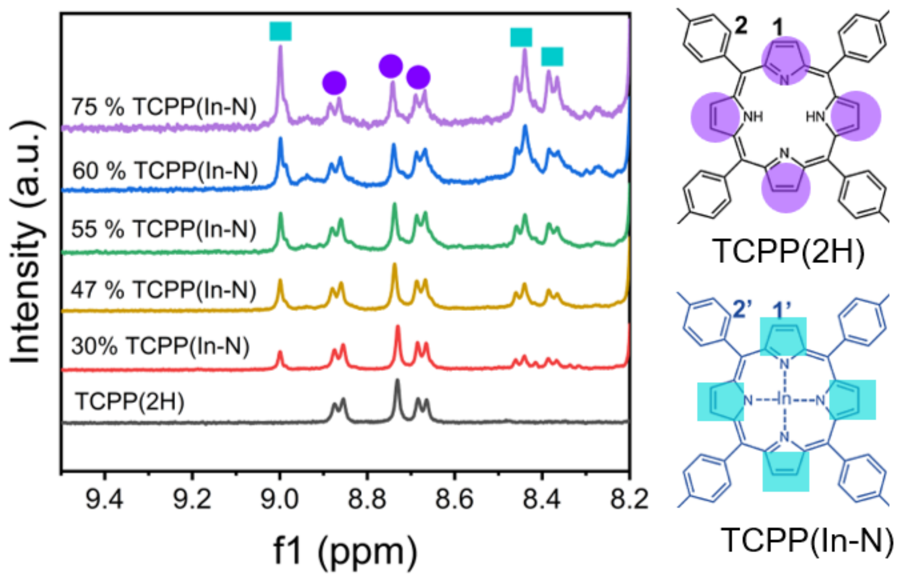

Supplementary Fig. 11. ^{1}HNMR. ^{1}H NMR spectra of M68N@In-TCPP composites with percentage of metallated TCPP(In) in In-TCPP. The location of the H atom is labeled in the molecular structure of the TCPP and TCPP(In). The values of 30~75% are the percentage of metallated TCPP(In). Source data are provided as a Source Data file.

The disappearance of N-H signal upon coordination with In implies porphyrin ring coordination with In. A detailed comparison of ^{1}H NMR spectra between TCPP-based MOFs and TCPP alone is presented in Supplementary Fig. 11. The spectra of acid-digested M68N@In-TCPP exhibit two sets of signals corresponding to free-base porphyrin (TCPP) and indium-metalated porphyrin (TCPP(In)). The peak at 8.61 ppm represents the eight hydrogen atoms of the pyrrole in the TCPP linker (H-1). In the ^{1}H NMR spectra, the doublet peaks at 8.75 and 8.57 ppm correspond to the outer aromatic hydrogens (H-2)[8,9]. The percentage of metallated TCPP(In) was determined by integrating the *β*-pyrrole peaks of metalated porphyrin (H-1′, 9.0 ppm, s, 8H) and unmetalled porphyrin (H-1, 8.75 ppm, s, 8H) using the following equation[9,10]:

$$C_{\text{In-N}}\ \% = S_{\text{In-N-9.0}}/(S_{\text{In-N-9.0}}+S_{\text{2H-8.73}}) \times 100\ \% \tag{2}$$

where C is the percentage of metallated TCPP(In); S is the area of *β*-pyrrole peak in

porphyrin.

By adjusting the metal-to-linker ratio, the In-N content can be varied from 30% to 70%. ICP-MS results show a strong correlation between the indium concentration in the porphyrin ring and its mass content in M68N@InTCPP (Supplementary Table 2).

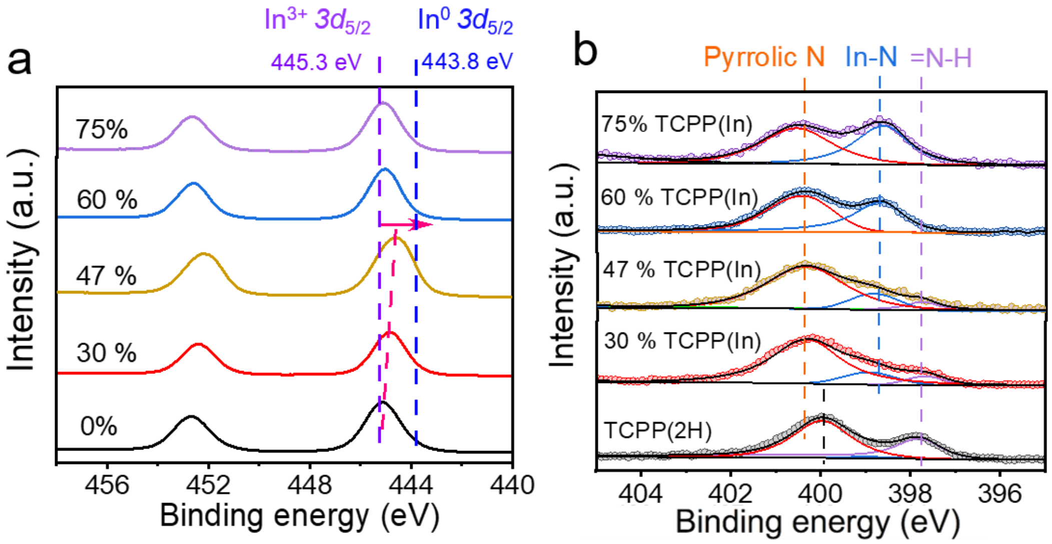

Supplementary Fig. 12. XPS. The XPS spectra of M68N@In-TCPP with different content of indium-metallated porphyrin (30 % - 75 %), **a** In *3d*, **b** N *1s*. Source data are provided as a Source Data file.

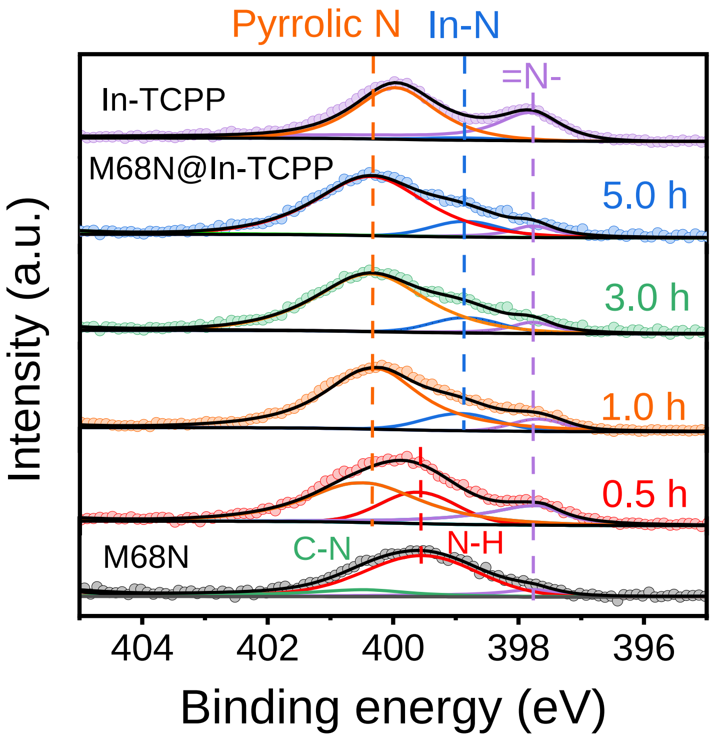

Supplementary Fig. 13. XPS. XPS spectra of N *1s* in M68N, In-TCPP, M68N@In-TCPP samples. Source data are provided as a Source Data file.

The N *1s* XPS spectrum of M68N can be deconvoluted into two peaks located at 397.8 and 399.6 eV, corresponding respectively to C–N and N–H of NH_2-BDC ligand. For In-TCPP, the two main peaks observed at 397.8 eV and 400.1 eV are assigned to uncoordinated N and porphyrin center pyrrolic N, respectively. Compared to pure In-TCPP, the additional peak at 398.9 eV of M68N@In-TCPP is likely attributed to N atoms bound to indium sites, accompanied by a 0.3 eV upshift due to the formation of an In-N bond. Notably, the N *1s* spectra of the reaction at 0.5 h showed both the peaks of amine and pyrrolic N, because the TCPP was initially adsorbed on the surface of M68N with relatively low contents. After that, the peak of N *1s* upshifted to higher binding energy after 1 h of reaction. The In-N bond was newly formed at 398.9 eV and then became obvious with increasing the reaction time to 5 h. The ratios of In/N atoms were also affected by the growth process, resulting in the formation of surface defects of In nodes in the M68N@In-TCPP hybrid.

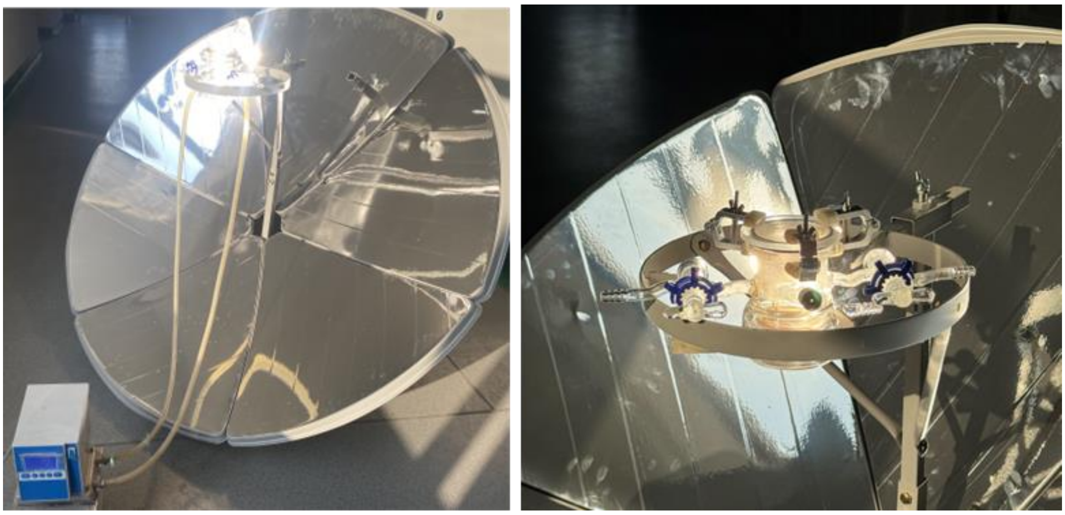

Supplementary Fig. 14. Photograph of reaction equipment. Photographs of the photocatalytic reaction equipment for coupled CO_2 reduction and H_2O oxidation under natural sunlight at HUST campus using M68N@In-TCPP photocatalyst.

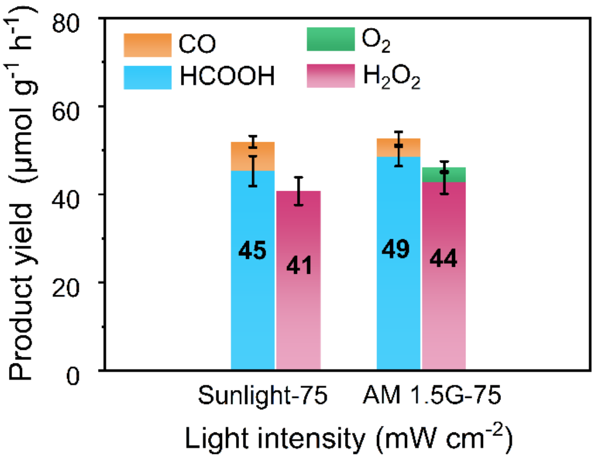

Supplementary Fig. 15. CO_2 photoreduction. The photocatalytic performance of M68N@In-TCPP using different light sources: sunlight irradiation with a light intensity of 75 mW cm^{-2} and AM 1.5 G (75 mW cm^{-2}). The experimental error bars represent the standard deviations of three independent measurements. Source data are provided as a Source Data file.

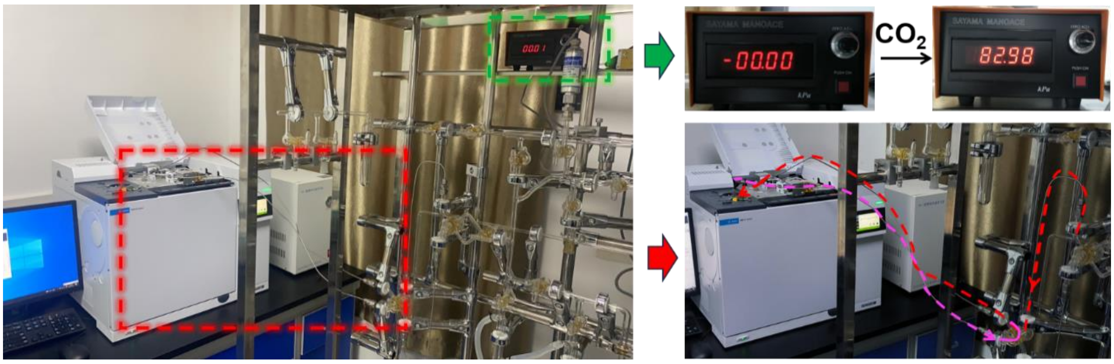

Supplementary Fig. 16. Photograph of reaction equipment. The system used for photocatalytic CO_2 reduction operates within a closed gas circulation setup. Inside the green box is a pressure gauge that monitors the system's pressure, which can be set to zero before introducing CO_2 gas (82.98 kPa). The red box contains the gas detection pathway for online sampling. The purple line represents the carrier gas (Ar), which mixes with the product gas from the red pathway before being injected into the GC for analysis.

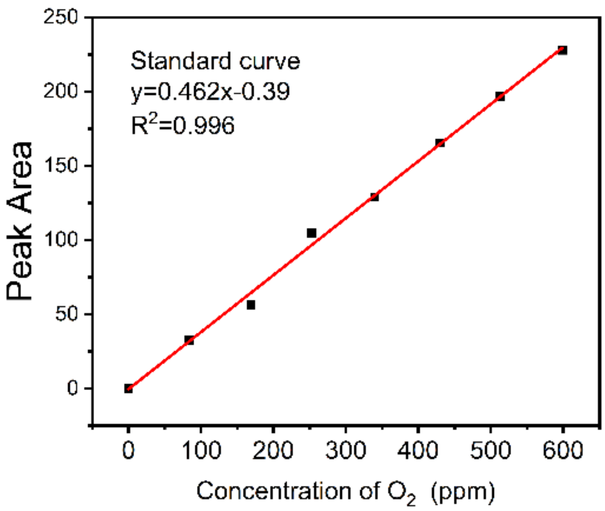

Supplementary Fig. S17. Product determination. The standard curve of detecting O_2 by the GC (Agilent-8860, USA). Source data are provided as a Source Data file.

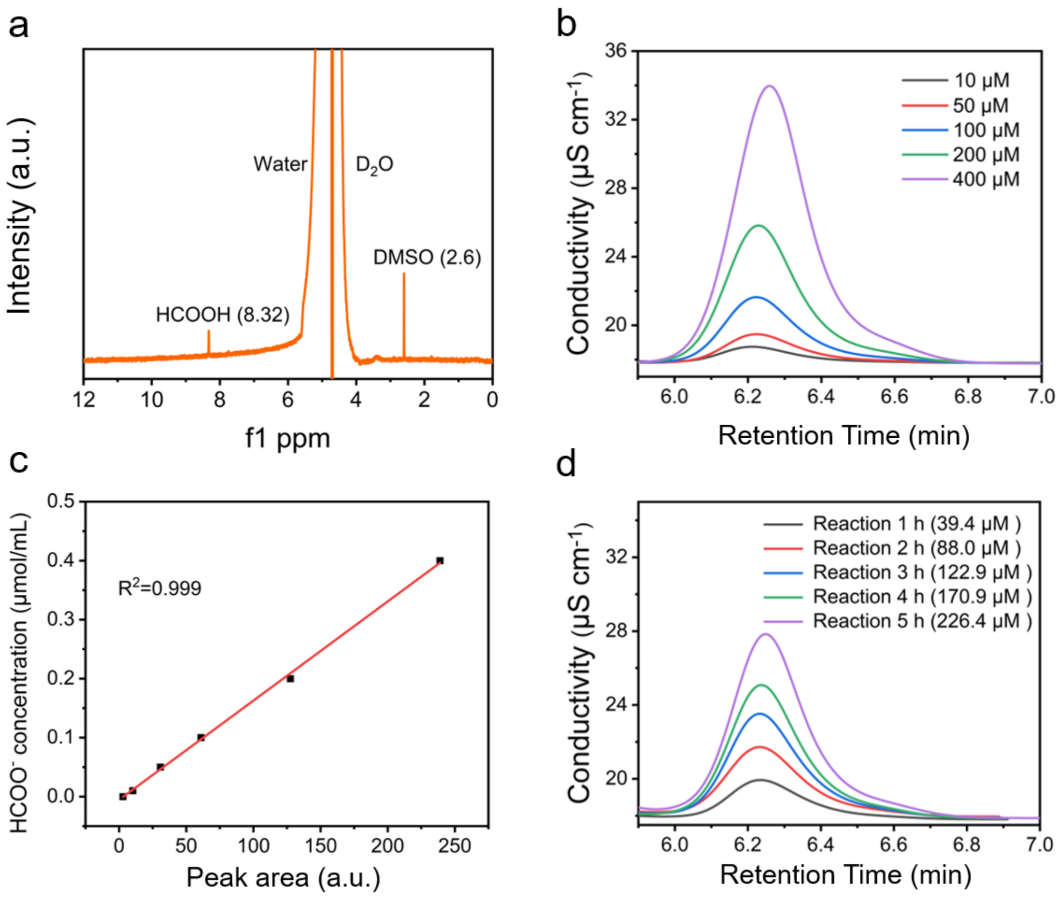

Supplementary Fig. 18. Product determination. Quantitative determination of HCOOH production. **a** ^1H NMR spectrum of liquid products after 5 h of photocatalytic reaction. **b** Ion chromatograph (IC) patterns of $HCOO^-$ solution (10-400 μM). **c** Standard curve of $HCOO^-$ solution (10-400 μM) by IC measurement. **d** $HCOO^-$ concentration at the given time of photocatalytic reaction by the standard curve. Source data are provided as a Source Data file.

The liquid products in the photocatalytic reaction were monitored by ^1H NMR. The peak at 8.32 ppm in the ^1H NMR spectrum was recognized as HCOOH. Therefore, the evolution rate of the HCOOH product was detected by IC.

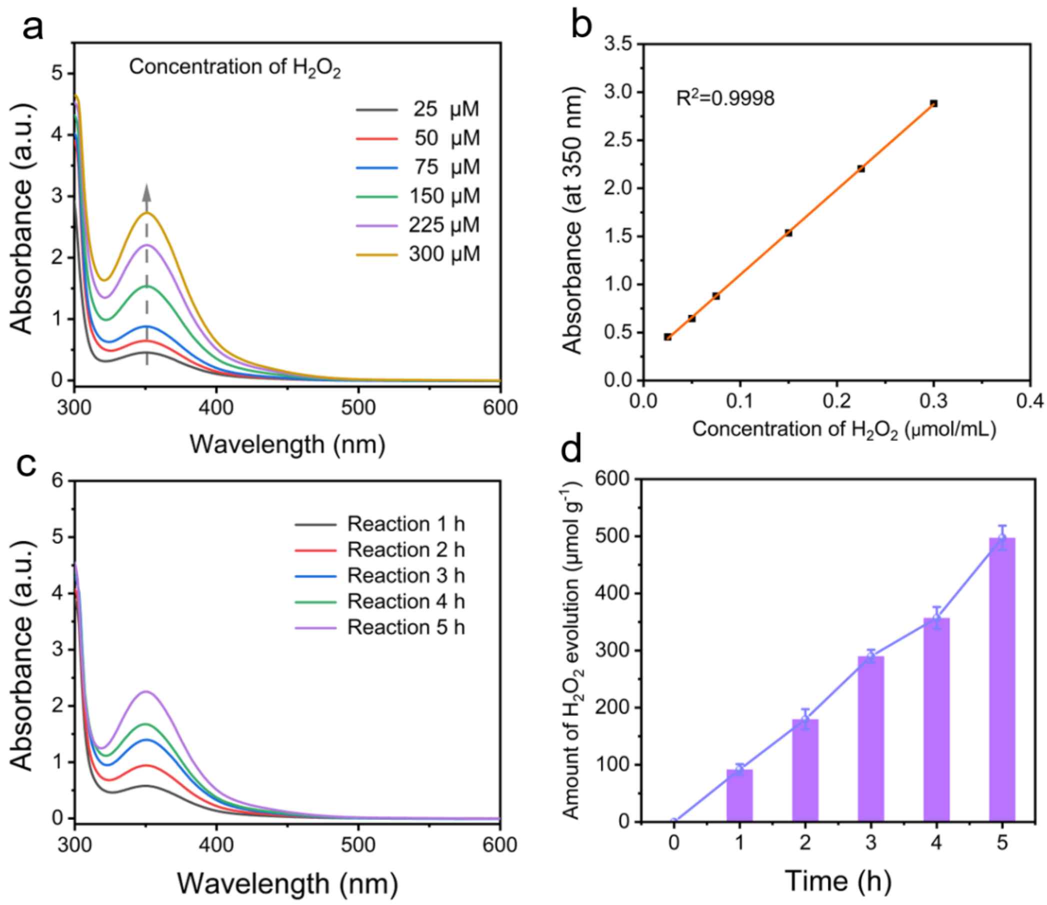

Supplementary Fig. 19. Product determination. Quantitative determination of H_2O_2 production by colorimetry method: $H_2O_2 + 3I^- + 2H^+ \rightarrow I_3^- + 2H_2O$. **a** UV-Vis spectra of H_2O_2 solution with different concentration in $C_8H_5KO_4$/KI solution. **b** Standard curve of H_2O_2 solution with different concentration at λ = 350 nm. **c** Light absorption of liquid reaction system at the given time of photocatalytic reaction using colorimetry method. **d** Quantitative determination of H_2O_2 production at given time interval by the standard curve. The experimental error bars represent the standard deviations of three independent measurements. Source data are provided as a Source Data file.

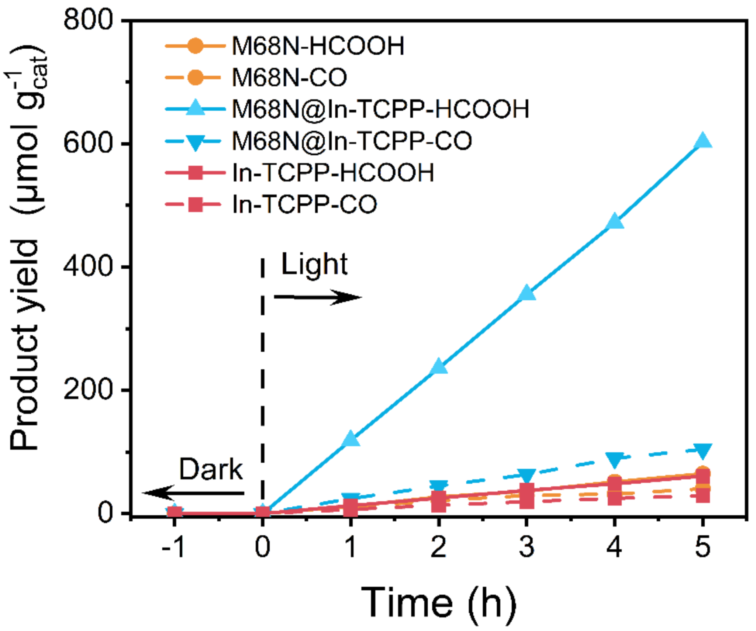

Supplementary Fig. 20. **CO_2 photoreduction.** Time courses of HCOOH and CO evolution by photocatalytic CO_2 reduction over various photocatalysts under visible light ($\lambda \geq 400$ nm). Source data are provided as a Source Data file.

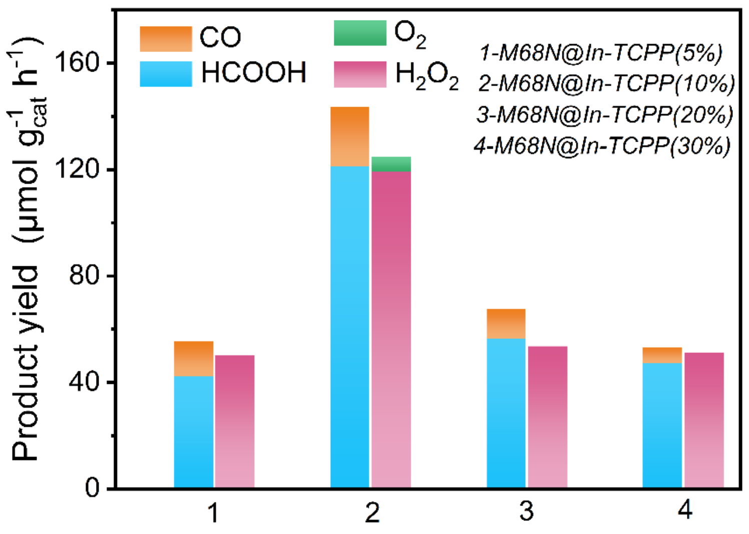

Supplementary Fig. 21. CO_2 photoreduction. Time courses of product evolution by photocatalytic CO_2 reduction of M68N@In-TCPP with different TCPP molar ratios (5% to 30%) under visible light ($\lambda \geq 400$ nm). Source data are provided as a Source Data file.

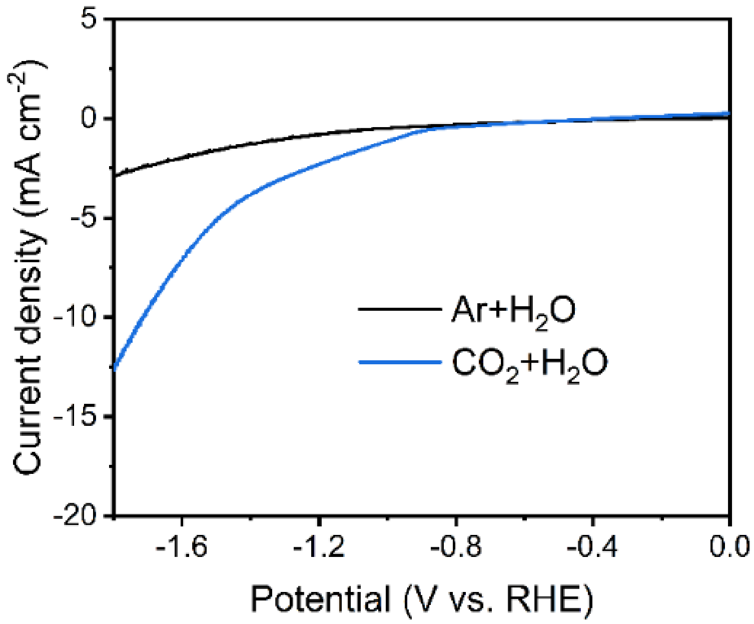

Supplementary Fig. 22. Linear sweep voltammetry curve. The Linear sweep voltammetry (LSV) curve of M68N@In-TCPP in CO_2 and Ar atmosphere with KOH (0.1 M) solution. Source data are provided as a Source Data file.

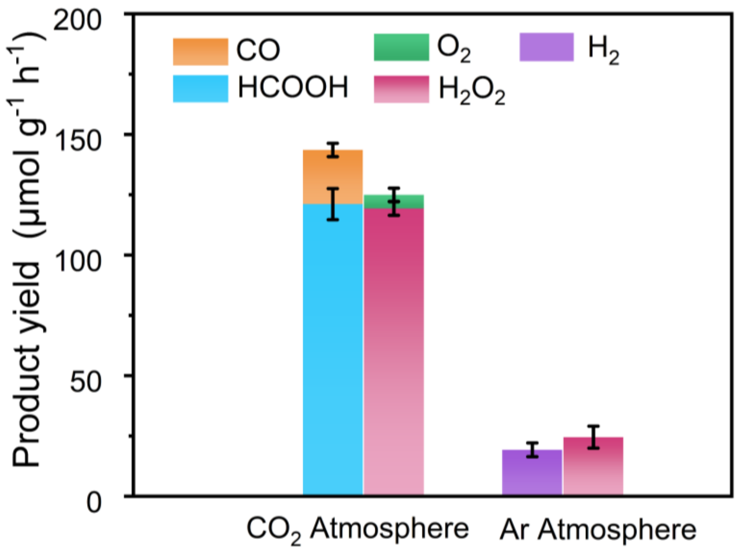

Supplementary Fig. 23. CO_2 photoreduction. The HCOOH, CO, H_2, and H_2O_2 production rates of the control experiments under different atmospheres with M68N@In-TCPP. The experimental error bars represent the standard deviations of three independent measurements. Source data are provided as a Source Data file.

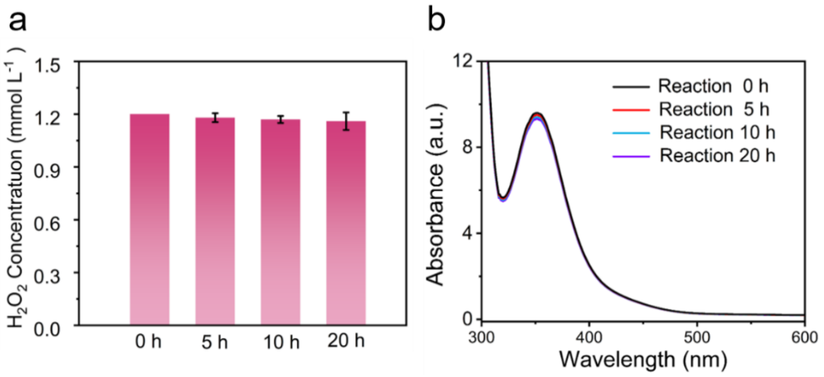

Supplementary Fig. 24. CO_2 photoreduction and UV adsorption spectra. Changes in H_2O_2 concentration after introducing H_2O_2 (1.2 mM) into the photocatalytic system with M68N@In-TCPP in Ar. H_2O_2 was dissolved in CH_3CN to avoid the interference by H_2O. **a** H_2O_2 concentration during light irradiation, **b** the corresponding UV adsorption spectra of I_3^- in the presence of H_2O_2. The experimental error bars represent the standard deviations of three independent measurements. Source data are provided as a Source Data file.

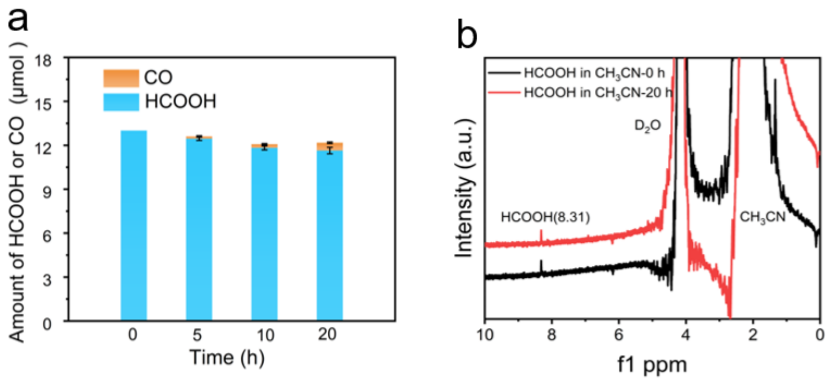

Supplementary Fig. 25. CO_2 photoreduction and ^{1}H-NMR. Changes in HCOOH concentration after introducing HCOOH (1.3 mM) into the system with M68N@In-TCPP in Ar. HCOOH was dissolved in CH_3CN to avoid the interference by H_2O. **a** HCOOH concentration during light irradiation, **b** the corresponding ^{1}H-NMR signal of the solution. The experimental error bars represent the standard deviations of three independent measurements. Source data are provided as a Source Data file

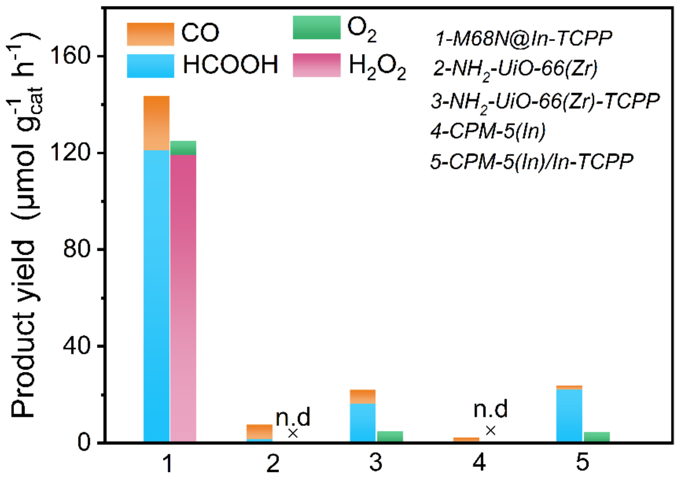

Supplementary Fig. 26. CO_2 photoreduction. Comparison of product evolution rates of M68N@In-TCPP by the reported related MOFs. Source data are provided as a Source Data file.

NH_2-UiO-66 (Zr) with same NH_2-BDC ligand and In-carboxylate framework (replace linker NH_2-BDC to 1,3,5-benzenetricarboxylate (BTC), CPM-5(In), both of which have been reported to show no ability for H_2O-to-H_2O_2 oxidation[11,12].

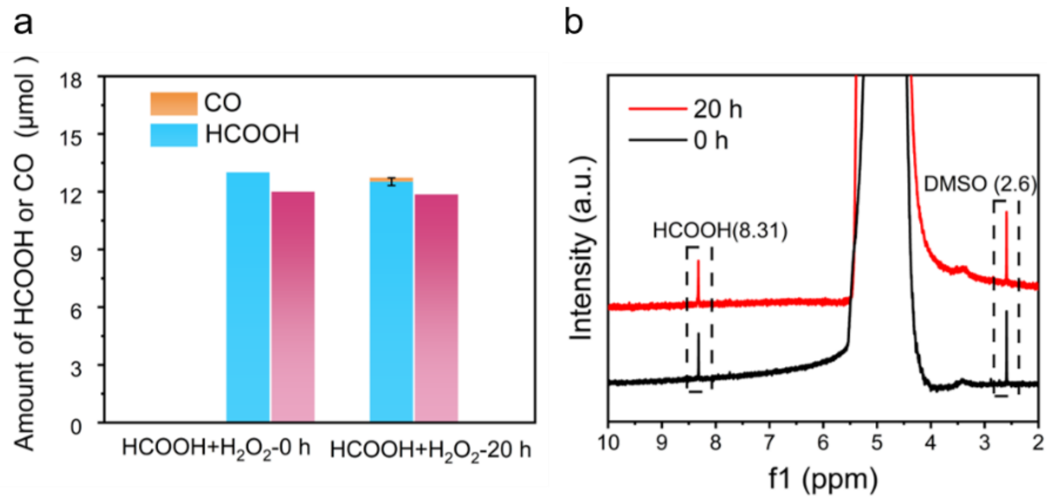

Supplementary Fig. 27. CO_2 photoreduction and ^{1}H-NMR. a Changes in HCOOH and H_2O_2 concentration under light irradiation, **b** the corresponding ^{1}H-NMR spectra of the solution. The experimental error bars represent the standard deviations of three independent measurements. Source data are provided as a Source Data file.

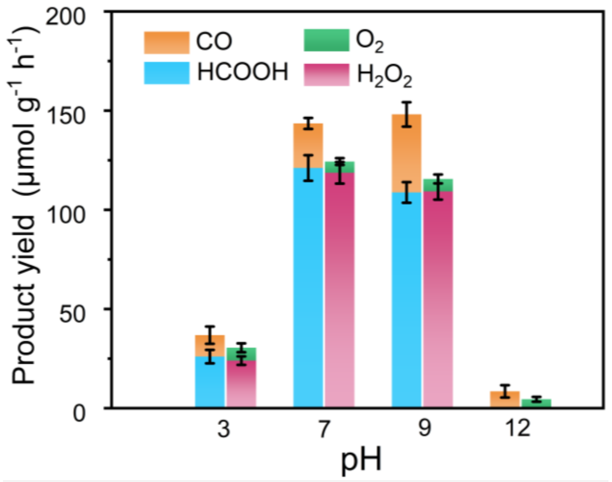

Supplementary Fig. 28. CO_2 photoreduction. The photocatalytic performance of M68N@In-TCPP at different pH. The pH of the reaction solution is adjusted by adding the NaOH or HCl (1 M). The experimental error bars represent the standard deviations of three independent measurements. Source data are provided as a Source Data file.

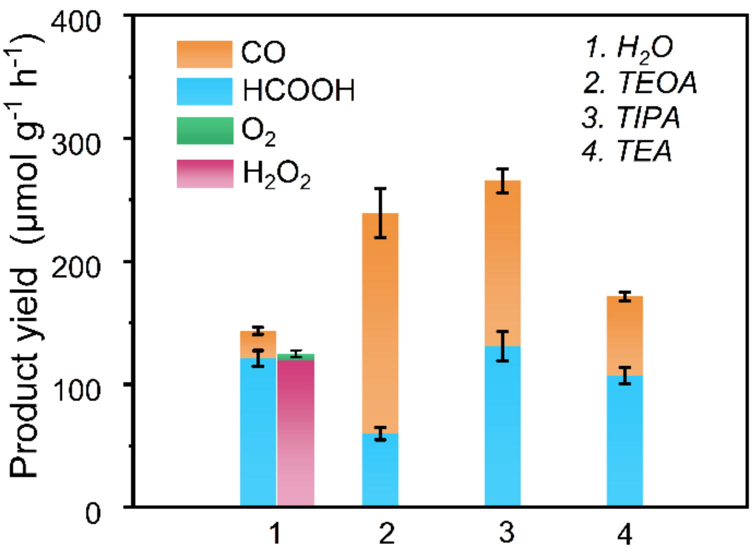

Supplementary Fig. 29. CO_2 photoreduction. Comparison of electron donors for the photocatalytic performance of M68N@In-TCPP. The sacrificial agents are mixed with CH_3CN (acetonitrile). The volume ratio of sacrificial agent to CH_3CN is 1:4. TEOA: triethanolamine; TIPA: triisopropanolamine; TEA: triethylamine. The experimental error bars represent the standard deviations of three independent measurements. Source data are provided as a Source Data file.

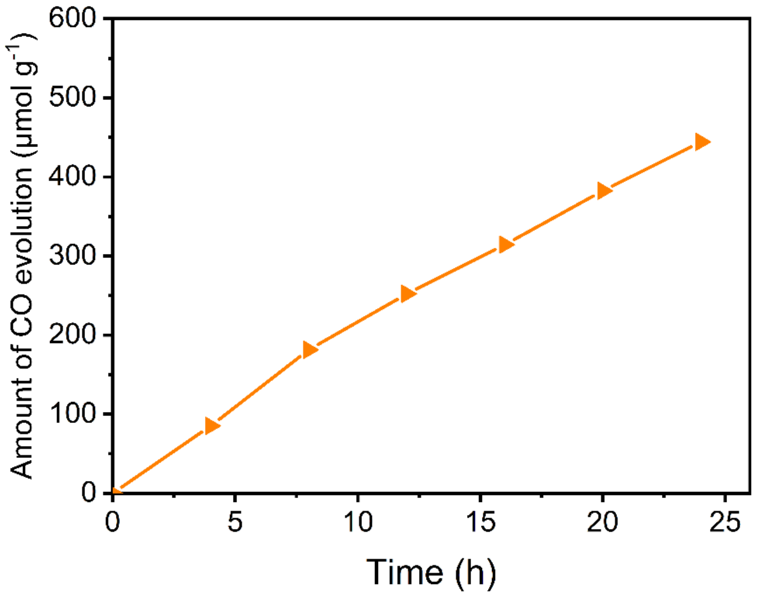

Supplementary Fig. 30. CO_2 photoreduction. Long-term test of CO production from photocatalytic CO_2 reduction. Source data are provided as a Source Data file.

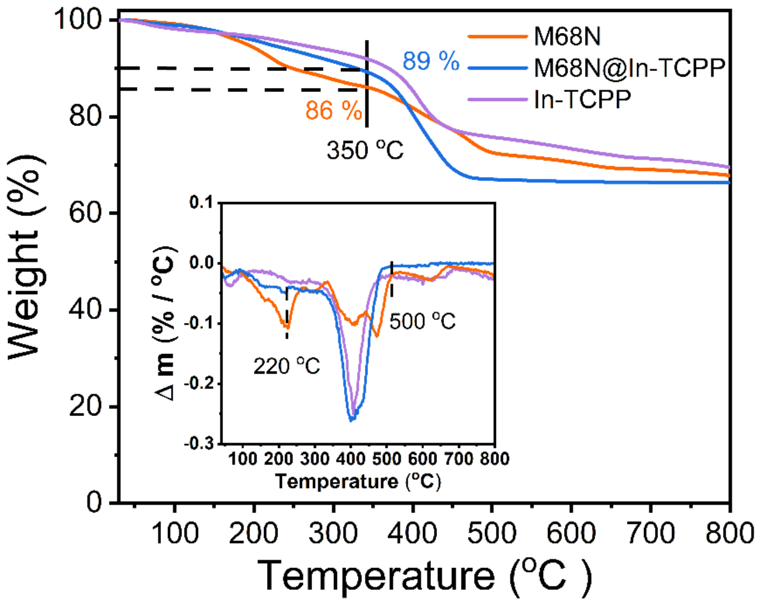

Supplementary Fig. 31. TGA. Thermogravimetric analysis (TGA) profiles of M68N, In-TCPP and M68N@In-TCPP with degassing before adsorption saturation H_2O. Source data are provided as a Source Data file.

TGA shows that M68N@In-TCPP is 4.0 wt % higher than that of M68N in the range of 150 to 350 °C due to the defective In-O sites in the heterostructure interface. And exhibits a similar loss weight tendency of In-TCPP during this stage, indicating that outer In-TCPP would enhance the stability of M68N@In-TCPP. The M68N shows a quickly low weight rate at 220 °C due to the decomposing of the NH_2 group, and all samples entirely converted to In_2O_3 around 500 °C. The weight loss of M68N@InTCPP over 350 °C belongs to the formation of In_2O_3. The higher weight loss of M68N@In-TCPP than that of M68N is attributed to the lower weight ratio of M68N in M68N@In-TCPP (90%).

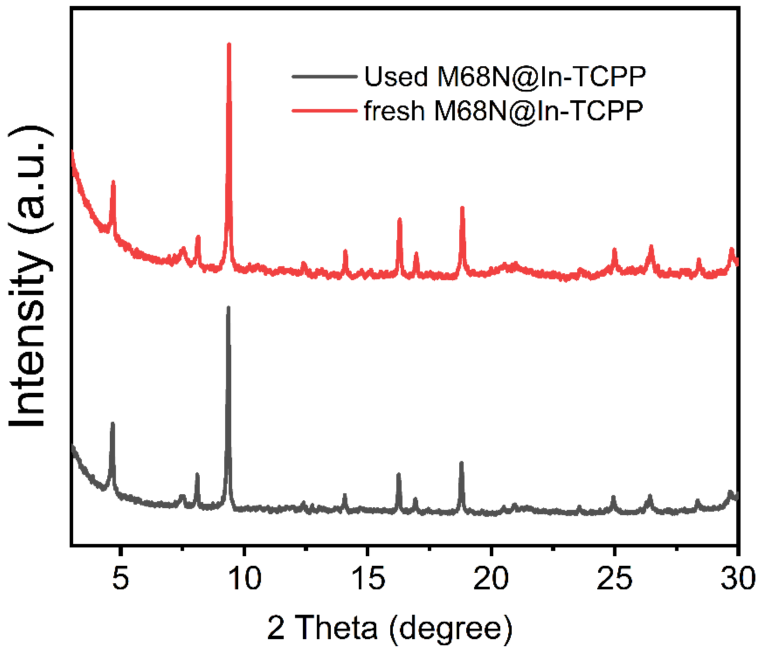

Supplementary Fig. 32. PXRD. PXRD patterns of fresh and used M68N@In-TCPP photocatalyst. Source data are provided as a Source Data file.

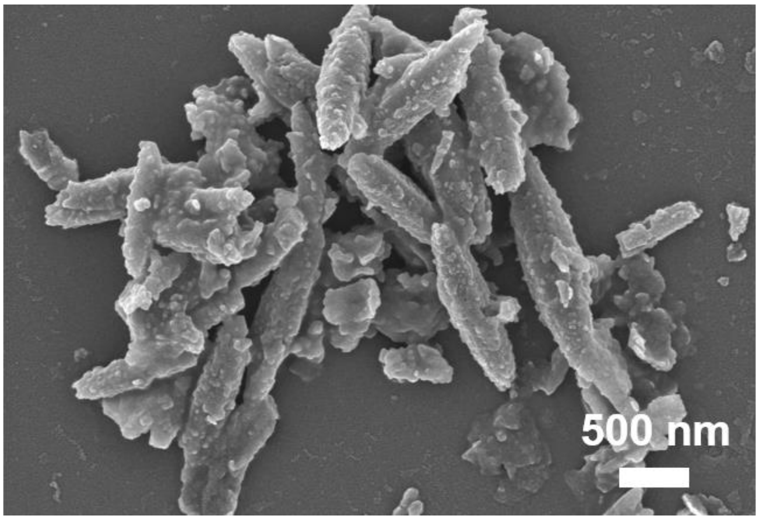

Supplementary Fig. 33. SEM. SEM image of M68N@InTCPP after the photocatalytic reaction.

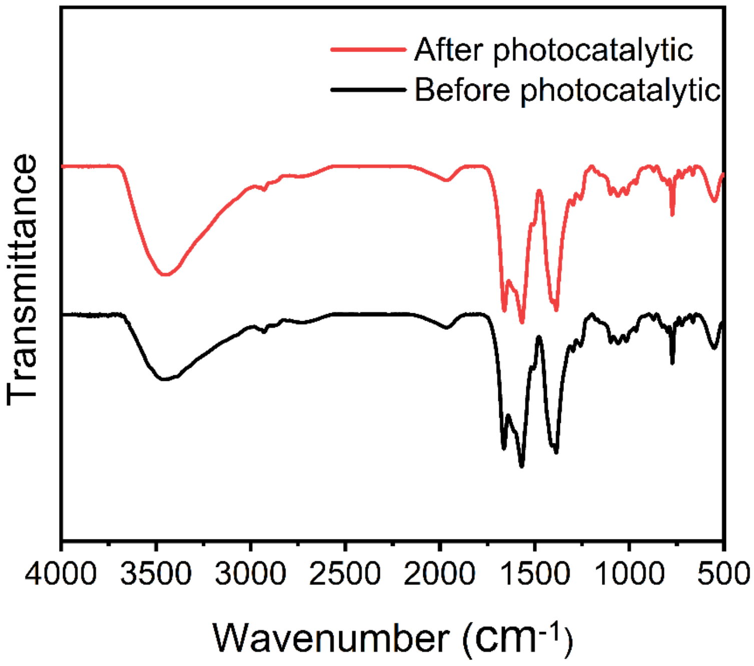

Supplementary Fig. 34. FT-IR. FT-IR spectra of M68N@In-TCPP before and after the photocatalytic reaction. Source data are provided as a Source Data file.

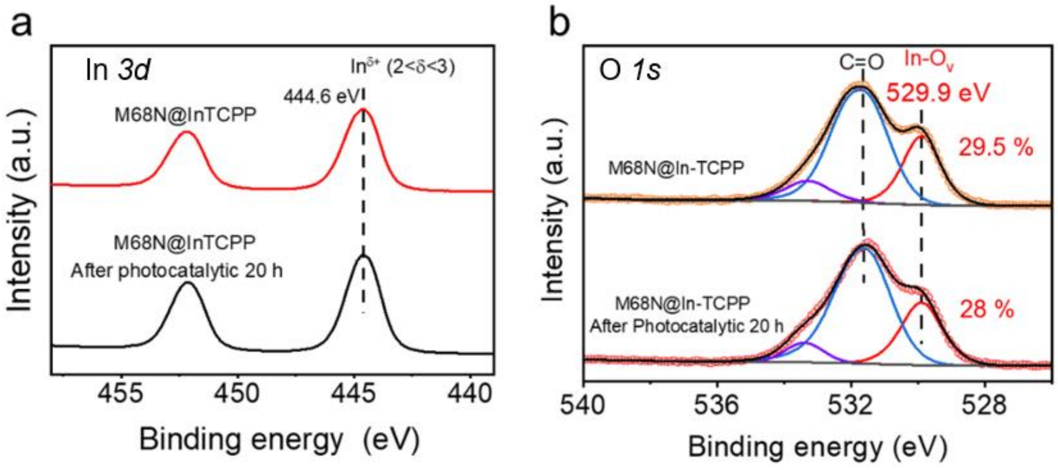

Supplementary Fig. 35. XPS. The **a** In *3d* and **b** O *1s* XPS spectra of M68N@InTCPP before and after the photocatalytic reaction. Source data are provided as a Source Data file.

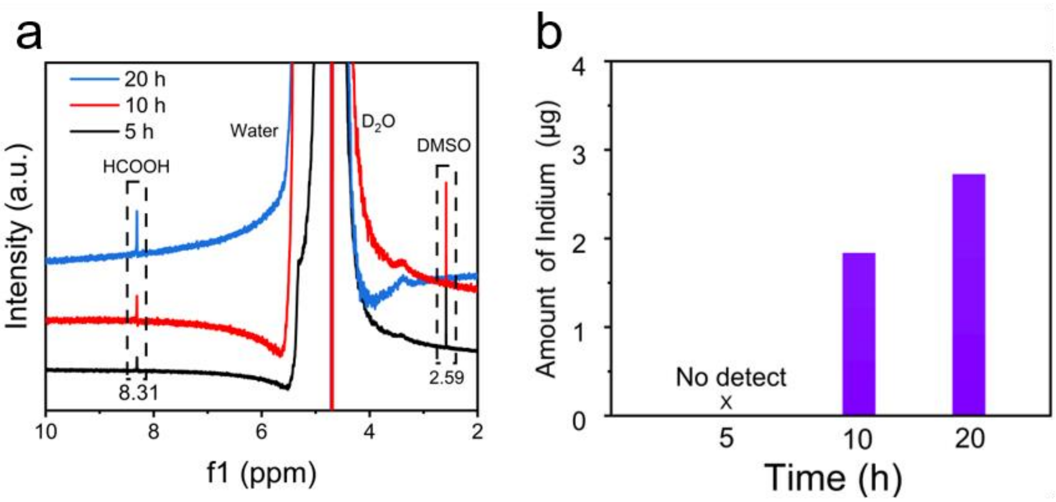

Supplementary Fig. 36. ^{1}H-NMR and ICP-MS results. a ^{1}H-NMR spectra of solution after 5, 10, and 20 h of reaction. The solution was collected by separating the M68N@In-TCPP for analysis. **b** The corresponding In content in the solution detected by Inductively Coupled Plasma Mass Spectrometer (ICP-MS). Source data are provided as a Source Data file.

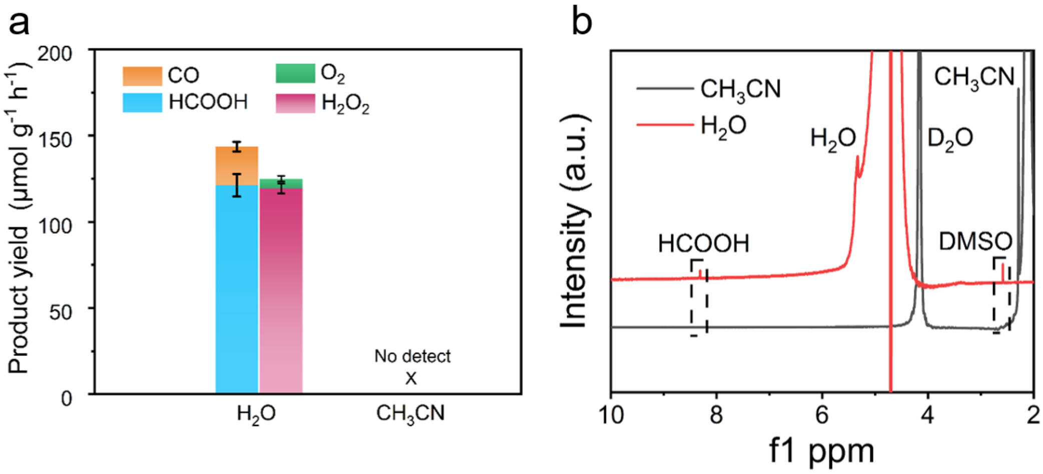

Supplementary Fig. 37. CO_2 photoreduction and ^{1}H-NMR. a Photocatalytic performance of M68N@In-TCPP in CH_3CN. **b** ^{1}H-NMR spectra of solution after 20 h of reaction. The experimental error bars represent the standard deviations of three independent measurements. Source data are provided as a Source Data file.

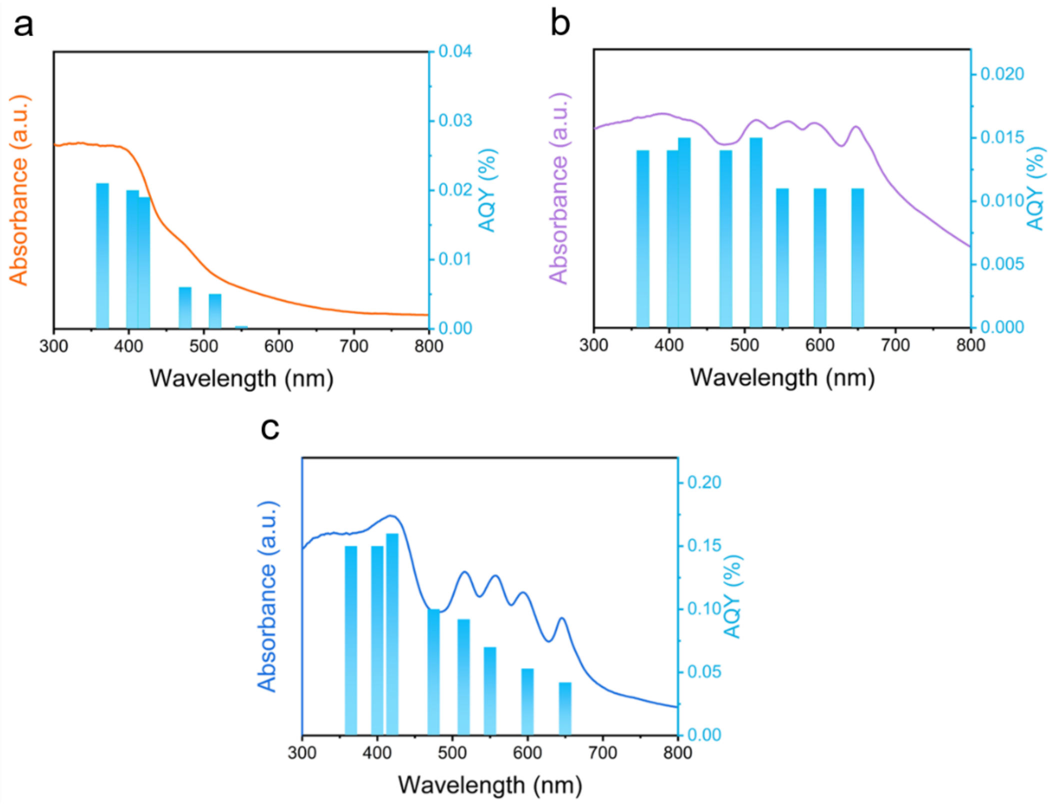

Supplementary Fig. 38. UV-visible diffuse reflectance spectra and AQY. Apparent quantum yield (AQY) values of **a** M68N, **b** In-TCPP and **c** M68N@In-TCPP photocatalysts under various wavelengths of light irradiation. The overlap is the UV-visible diffuse reflectance spectrum of each MOF photocatalyst. Source data are provided as a Source Data file.

The AQY of M68N was decreased with the extended wavelength of light irradiation, which is consistent with its UV-Vis absorption spectrum. In contrast, the changes in the AQY of In-TCPP at the range of 365~650 nm are not as significant as that of M68N due to the strong light absorption in the broad visible region. Thus, the formation of In-TCPP layer on the M68N surface results in excellent performance over M68N@In-TCPP under full visible region. The AQY of the three samples is listed in Supplementary Table 7. The detail calculation process of AQY value was listed as follows, take 420 nm for example: The catalyst was irradiated by a 420 nm

monochromatic light for 1 hour, where the average intensity of monochromatic light was calibrated to be 9.42 mW/cm^2, and illumination area (3.14 cm^2), respectively. The number of incident photons (*N*) was derived to be:

$$N = \frac{29.6 \times 10^{-3} \times 3600 \times 420 \times 10^{-9}}{6.626 \times 10^{-34} \times 3 \times 10^{8}} = 2.24 \times 10^{20} \quad (3)$$

The amount of CO and HCOOH molecules generated in 1 hour were 0.104 μmol and 0.182 μmol, respectively. Hence, the total consumed electron number (N_C) was derived to be: $N_c = (0.104 + 0.194) \times 2 \times 10^{-6} \times 6.02 \times 10^{23} = 3.584 \times 10^{17}$ Thus, the Apparent quantum yield (AQY) at 420 nm was calculated as:

$$AQY_{420nm} = \frac{3.584 \times 10^{17}}{2.24 \times 10^{20}} \times 100\% = 0.16\% \quad (4)$$

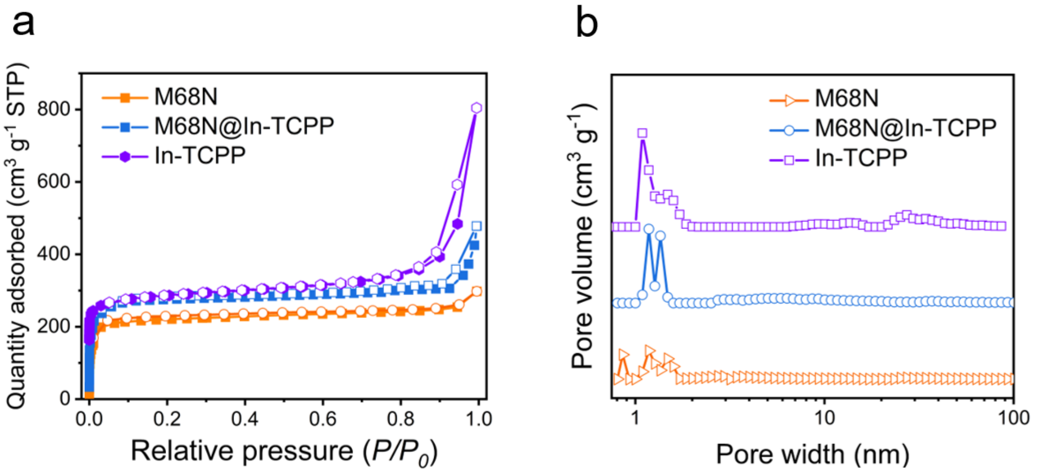

Supplementary Fig. 39. **N_2 adsorption−desorption isotherms and pore size distribution. a** N_2 adsorption−desorption isotherms and **b** pore size distributions of M68N, In-TCPP, M68N@In-TCPP. Source data are provided as a Source Data file.

The specific surface area of M68N@In-TCPP was enlarged from 675.9 to 798.4 m^2 g^{-1}. The pore size distribution shown in Supplementary Fig. 39b demonstrates the primary microporous property of the three MOFs, among which M68N@InTCPP displays the most regular microporosity with the dominant pore diameter centre at 1.2 and 1.4 nm. The narrow pore size distribution of hybrid MOFs suggests that the kinetic control of anisotropic growth results in the regular connection of metal with TCPP ligand on the M68N surface.

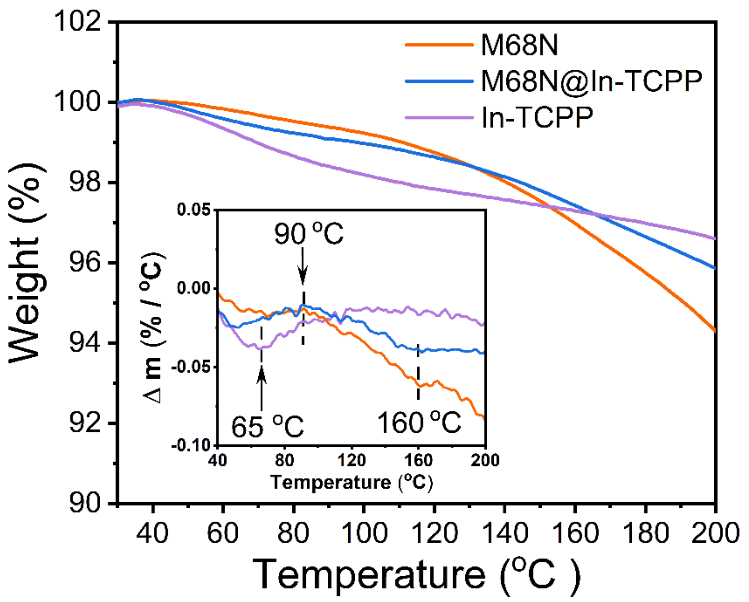

Supplementary Fig. 40. **TGA.** TGA of the samples, with degassing before adsorption saturation H_2O. The insert is the derivative thermogravimetry (DTG) curve. Source data are provided as a Source Data file.

TGA of the H_2O-saturated samples unveiled distinct desorption behaviors: M68N exhibited a two-stage desorption pattern, with water initially desorbing below 90°C and subsequently at elevated temperatures ranging from 160 to 200 °C. In contrast, water desorption from In-TCPP proceeded more readily, reaching a plateau at ~90 °C, indicative of a comparatively weaker interaction between H_2O and In-TCPP. Interestingly, the desorption from M68N@In-TCPP required higher temperatures (~160 °C) than In-TCPP, suggesting the pivotal role of M68N in H_2O adsorption of the heterostructure.

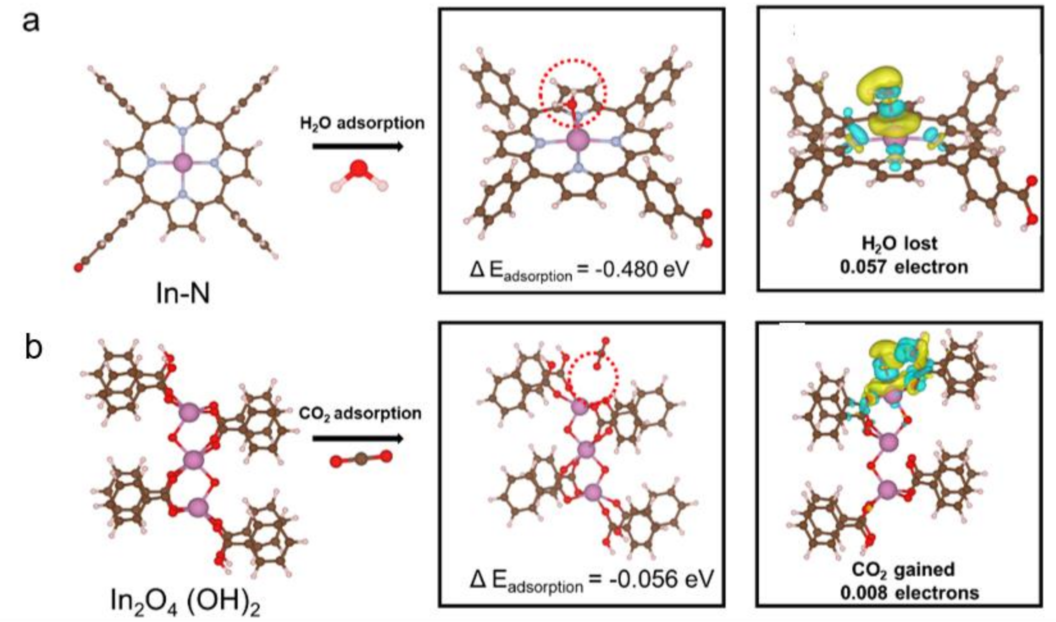

Supplementary Fig. 41. **DFT calculation.** Density function theory calculations on the charge difference density of H_2O adsorption at **a** In-N sites and CO_2 adsorption at **b** $InO_4(OH)_2$ sites. Source data are provided in Supplementary Data 1.

To analyze the adsorption performance of CO_2 and H_2O on $InO_4(OH)_2$ and In-N sites, we optimized the structures in which one of the In−O bonds between the node and modulator was broken and the adsorbed molecules such as CO_2 and H_2O were coordinated to the open indium site. Two theoretical pathways were proposed for reactant adsorption: one where CO_2 adsorbs at the In-N site and H_2O at $InO_4(OH)_2$ sites, and another with reversed adsorption (Supplementary Fig. 41). These calculations suggest that H_2O is more readily activated at $InO_4(OH)_2$ sites. For CO_2 adsorption on $InO_4(OH)_2$ sites, the adsorption energy was found to be -0.056 eV with an In-O distance of 3.38 Å (Supplementary Fig. 42). DFT calculation result reveals that H_2O adsorption energy is approximately -0.48 eV for both In-N and $InO_4(OH)_2$ sites. However, a notable elongation in the H-O bonds length of H_2O is from 0.972 to 0.979/0.983 Å at $InO_4(OH)_2$ and from 0.976 to 0.977 Å at In-N.

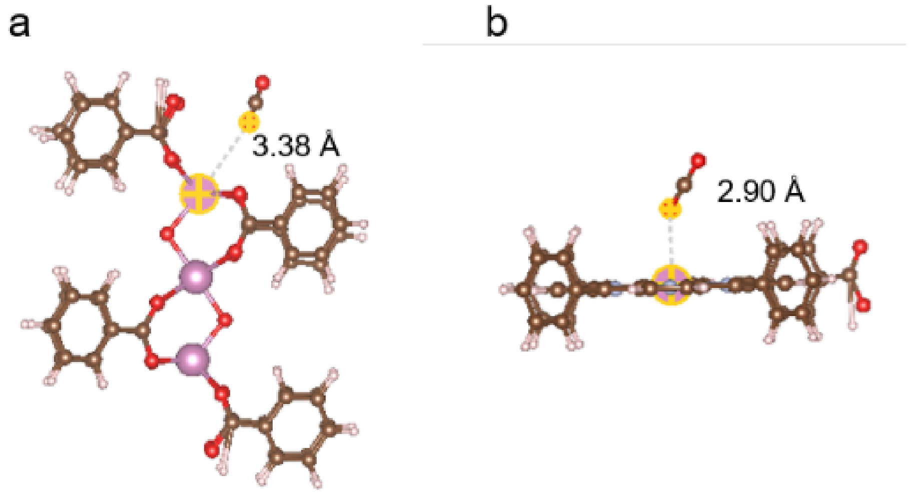

Supplementary Fig. 42. DFT calculation. DFT calculations of the distance between the adsorbed CO_2 and In atoms in **a** $InO_4(OH)_2$ and **b** In-N sites. Source data are provided in Supplementary Data 1.

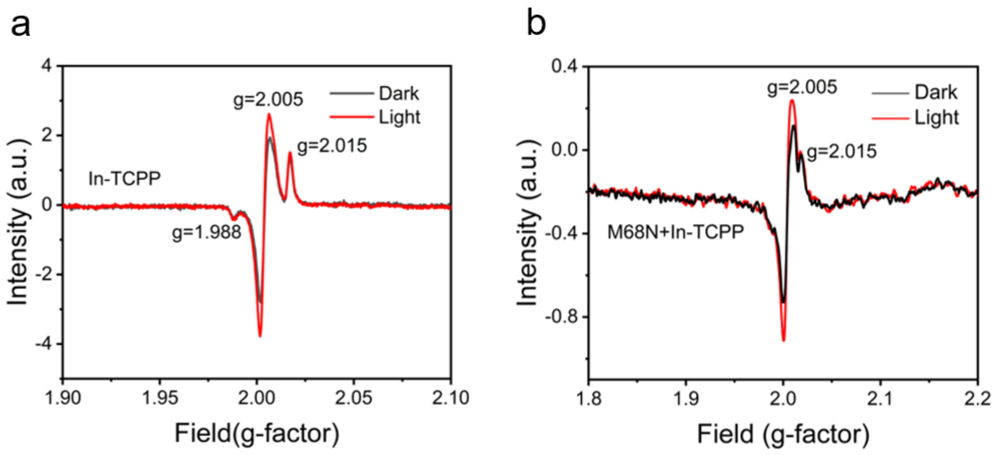

Supplementary Fig. 43. In-situ EPR. EPR spectra of **a** In-TCPP and **b** a mechanical mixture of M68N and In-TCPP (M68N+In-TCPP). Source data are provided as a Source Data file.

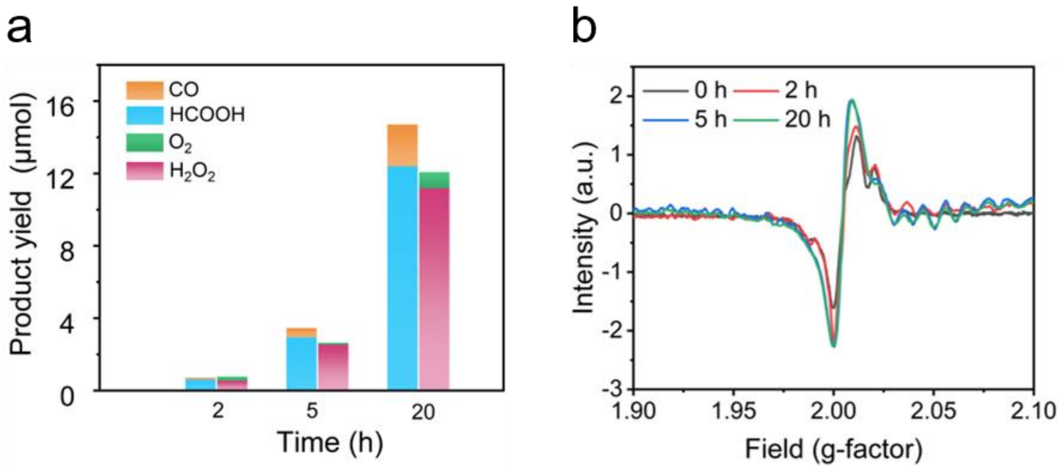

Supplementary Fig. 44. CO_2 photoreduction and In-situ EPR. a Photocatalytic performance of M68N@In-TCPP after reaction 2, 5, and 20 h. **b** The evolution of O_v within the M68N@In-TCPP heterostructures during the reaction. Source data are provided as a Source Data file.

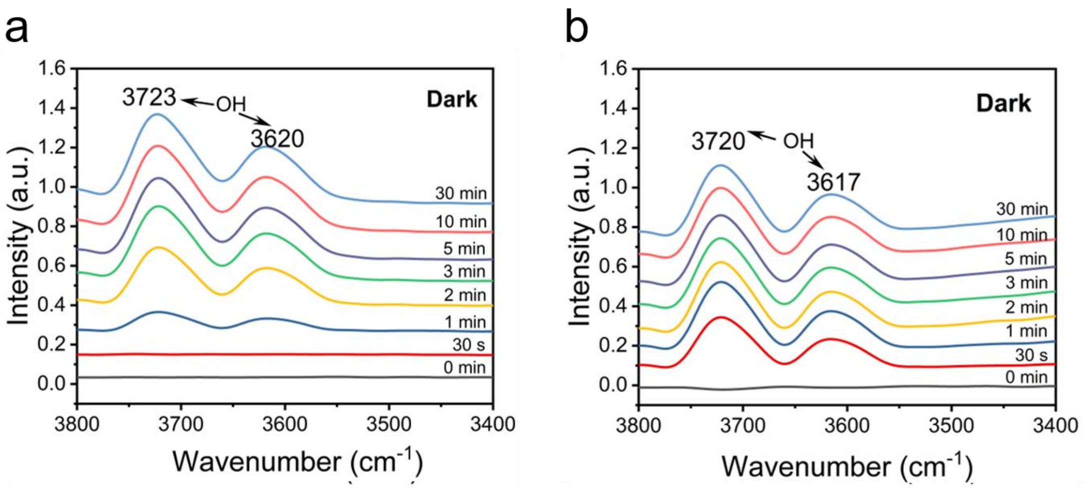

Supplementary Fig. 45. In situ DRIFTS. In situ DRIFTS tests in the region of 3400-3800 cm^{-1} on **a** M68N and **b** M68N@In-TCPP in the dark. Source data are provided as a Source Data file.

The DRIFTS spectra of both M68N and M68N@In-TCPP show broad peaks at 3723 and 3620 cm^{-1} owing to the stretching vibrations of surface OH of H_2O, respectively. The stronger signal of H_2O adsorption of the M68N at 3723 cm^{-1} than that of M68N@In-TCPP is ascribed to the hydrophilic -NH_2 groups of NH_2-BDC ligand.

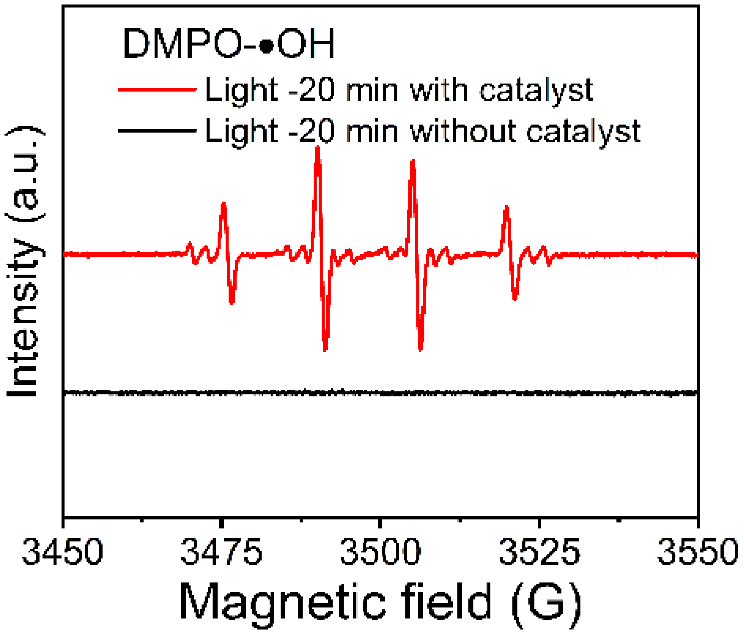

Supplementary Fig. 46. In-situ EPR. EPR spectra of DMPO-•OH with or without M68N@In-TCPP photocatalyst under light irradiation. Source data are provided as a Source Data file.

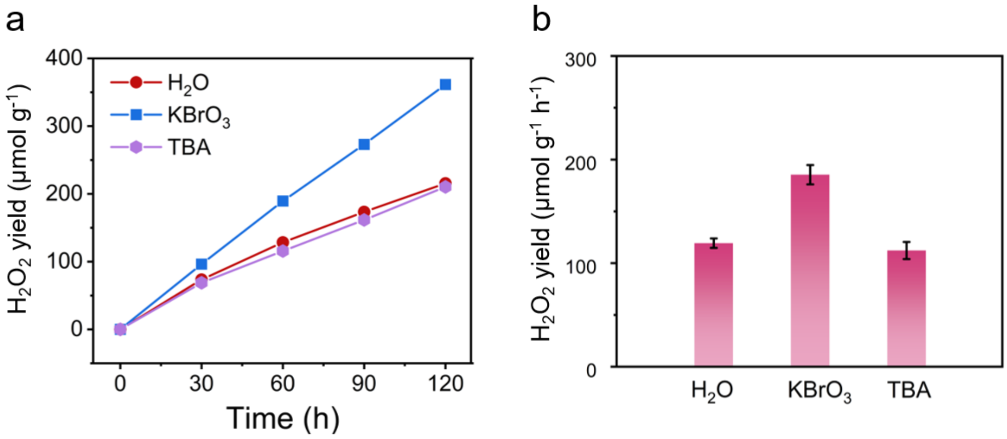

Supplementary Fig. 47. CO_2 photoreduction. The H_2O_2 yield over M68N@In-TCPP in CO_2 atmosphere with different sacrificial agents (10 mM $KBrO_3$, and TBA). **a** The H_2O_2 yield with reaction time. **b** The corresponding H_2O_2 production rate. The experimental error bars represent the standard deviations of three independent measurements. Source data are provided as a Source Data file.

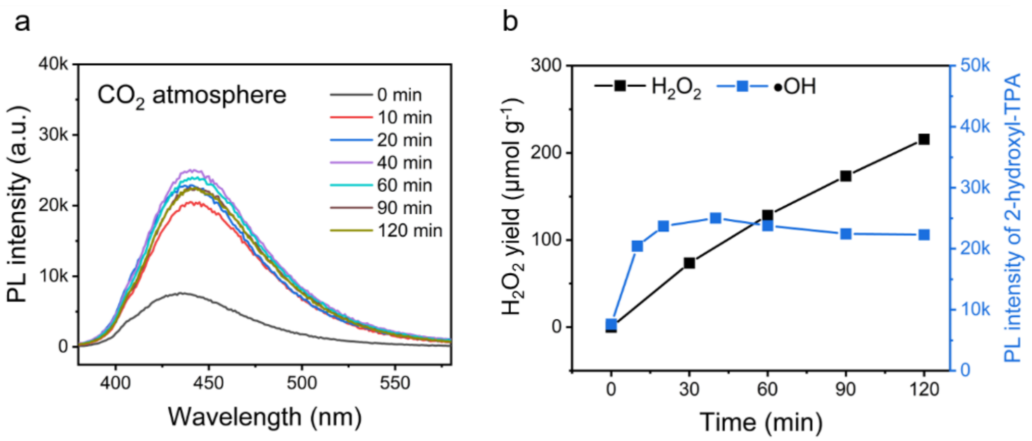

Supplementary Fig. 48. Photoluminescence spectra and CO_2 photoreduction. a Photoluminescence (PL) intensity of the reaction product between •OH radical and fluorescence probe of 2-hydroxy terephthalic acid (TPA) during the photocatalytic reaction. **b** The comparison in the change of •OH level to that of H_2O_2 yield during the photocatalytic reaction. Source data are provided as a Source Data file.

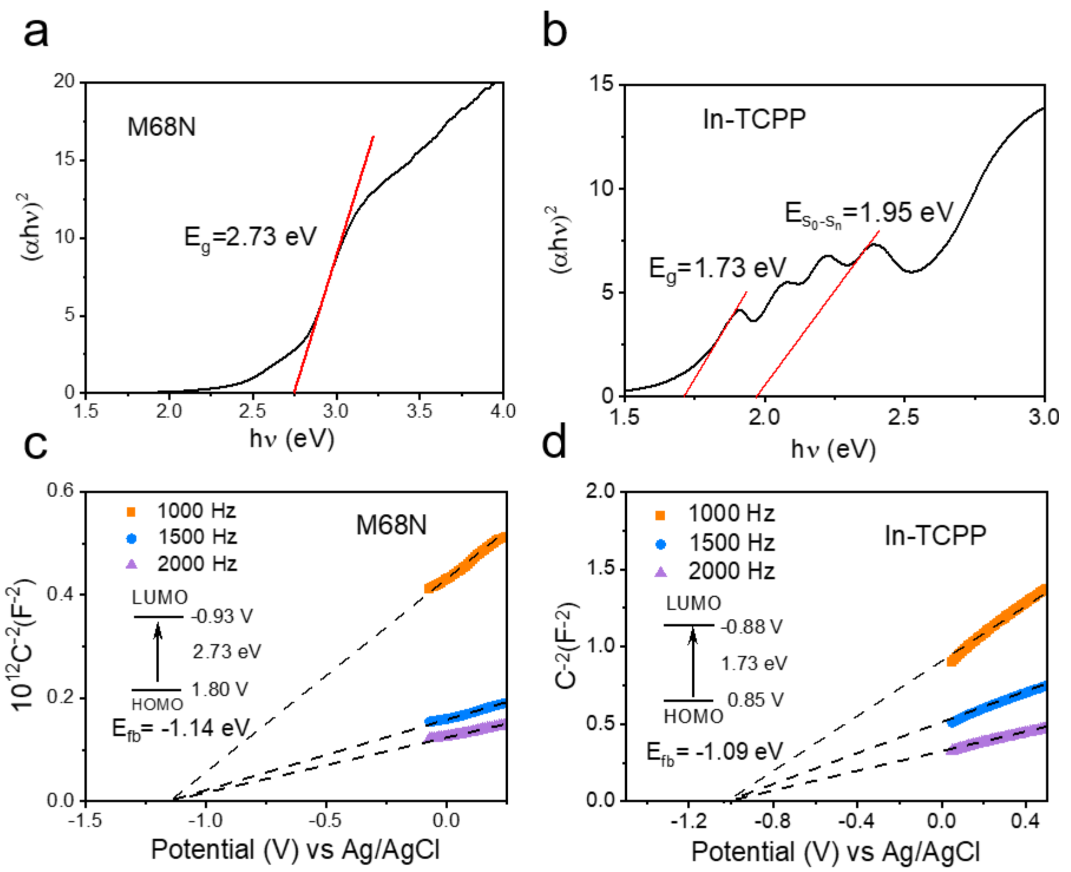

Supplementary Fig. 49. Mott-Schottky plots. Transformed Kubelka-Munk function plots of **a** M68N and **b** In-TCPP. Mott-Schottky plots of **c** M68N and **d** In-TCPP at different frequency. The inset presents the location of energy levels. Source data are provided as a Source Data file.

The energy levels versus normal hydrogen electrode (NHE) are calibrated by the difference of 0.2046 V between NHE and Ag/AgCl electrode.

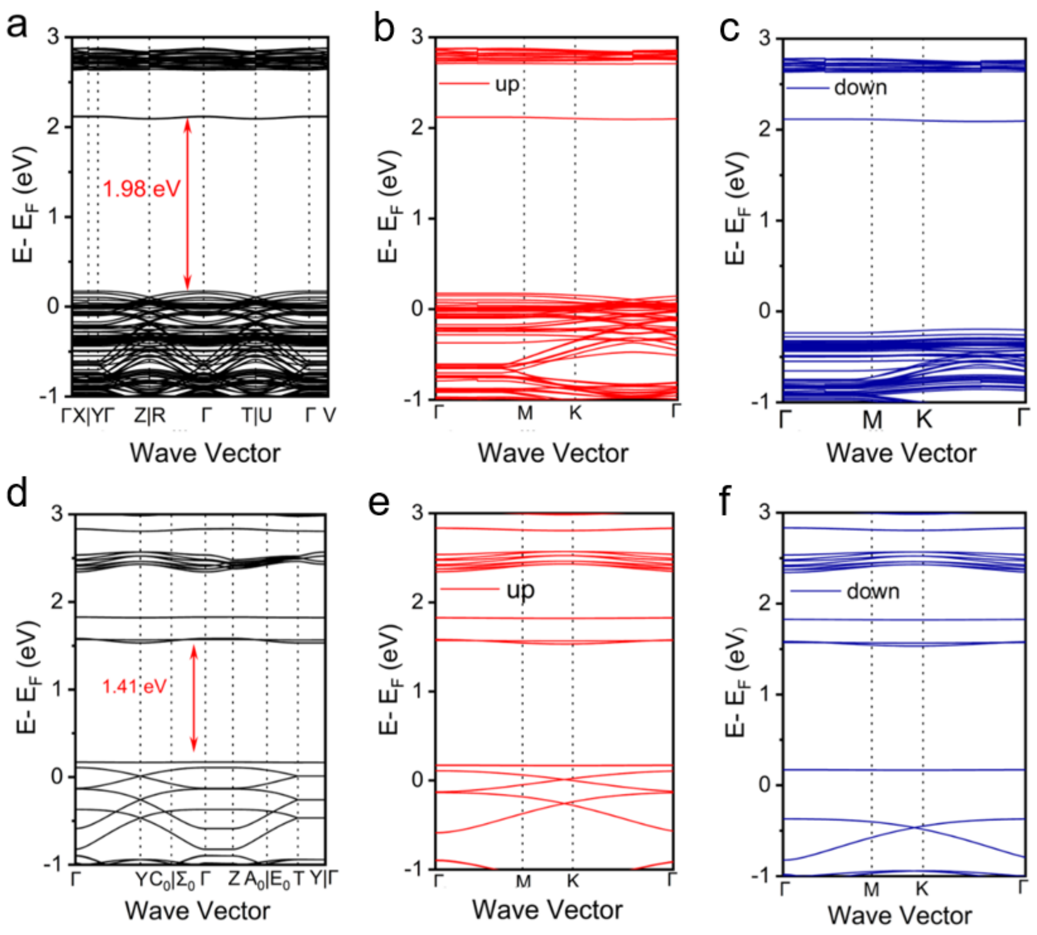

Supplementary Fig. 50. DFT calculation. The band structure of **a-c** M68N and **d-f** In-TCPP. Source data are provided as a Source Data file.

It can be seen that the M68N has a band gap of around 1.98 eV, which is larger than that of In-TCPP (1.41 eV). The top of the valence band near the Fermi level of M68N mainly originates from the C *2p* and O *2p* states of carboxyl and the bottom of the conduction band primarily stems from C *2p*, N *2p*, and In *3d* of the metal node. The top of the valence band near the Fermi level of In-TCPP is mainly dominated by the *2p* states of C (benzene rings) and O (In-O chains).

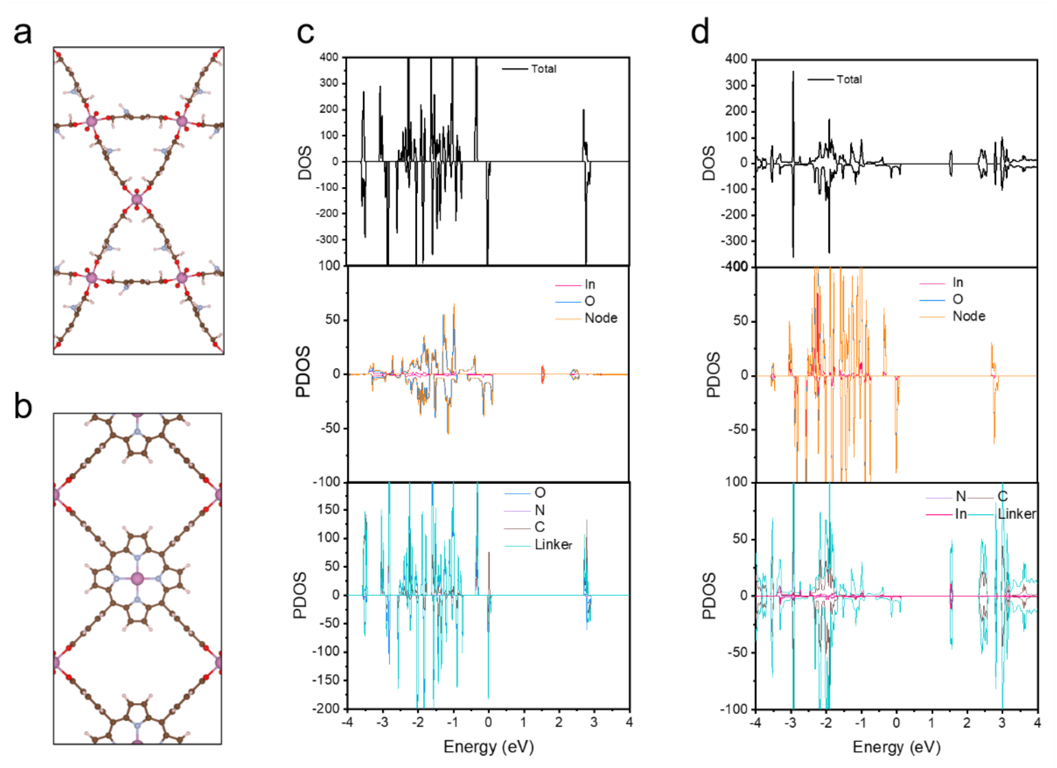

Supplementary Fig. 51. **DFT calculation.** The unit structure of **a** M68N and **b** In-TCPP. The corresponding density of state (DOS) and projected density of state (PDOS) of (c) M68N and (d) In-TCPP. Source data are provided as a Source Data file.

Density of state calculations indicated that In-carboxylic chains connected to metalloporphyrin significantly influence the HOMO of In-TCPP. In-TCPP primarily exhibits two photoexcitation modes: ligand-localized excitations characterized by π–π* transitions within metalloporphyrin and direct ligand-to-node excitations involving electron transfer from TCPP ligand to the $InO_4(OH)_2$ node. Ligand-localized excitations are notably more pronounced than the direct ligand-to-node transitions, leading to the predominance of the LUMO primarily to the metalloporphyrin units. The results suggest that the CO_2 reduction is likely facilitated at the metalloporphyrin sites, with M68N showing a superior capability for H_2O oxidation due to its more positive HOMO energy relative to In-TCPP.

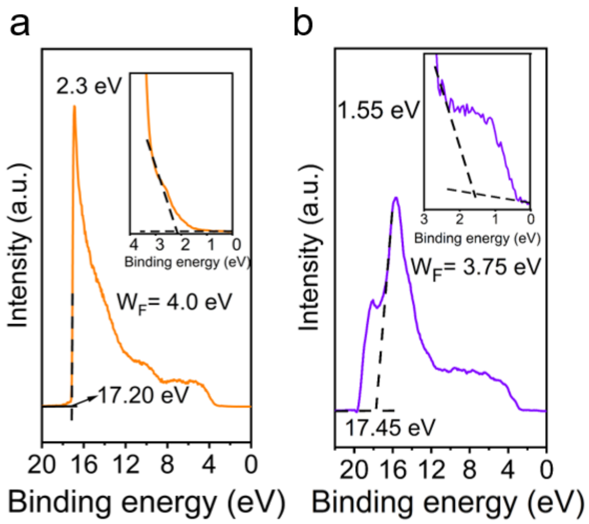

Supplementary Fig. 52. **Ultraviolet photoelectron spectra**. UPS spectra of **a** M68N and **b** In-TCPP. Source data are provided as a Source Data file.

For M68N, the work function (W_F) can be determined by the difference between the photon energy (21.2 eV) and the binding energy of the secondary cutoff edge.

$$\text{Equations: } W_F = (21.20 - E_{cut\text{-}off}) = 4.0 \text{ eV}. \quad (5)$$

The HOMO and LUMO of M68N can be determined by the equation:

$$E_{HOMO} \text{ (vs vacuum)} = -W_F - (E_F - E_{LUMO}). \quad (6)$$

The HOMO energy location is measured to be 2.3 eV below the potential of Fermi level (E_F), which corresponds to -6.30 eV and LUMO at -3.57 eV vs vacuum level based on the band gap of M68N (2.73 eV).

The HOMO and LUMO of M68N vs normal hydrogen electrode can be determined by the equation:

$$E_{HOMO} \text{ (vs NHE)} = -4.5 - E_{HOMO} \text{ (vs vacuum)}. \quad (7)$$

The HOMO and LUMO locations of M68N are 1.80 V and -0.93 V vs NHE.

Similarly, the W_F of In-TCPP is 3.75 eV vs vacuum level. The corresponding HOMO and LUMO locations of In-TCPP are measured at -5.35 eV and -3.62 eV vs vacuum level, and the HOMO and LUMO locations vs NHE are 0.85 V and -0.88 V.

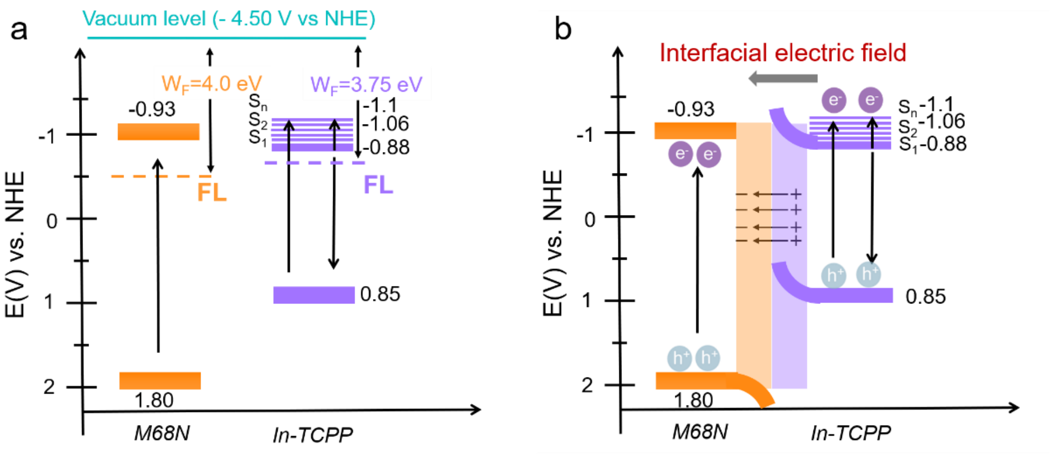

Supplementary Fig. 53. **Band structure. a** Energy locations of the lowest unoccupied molecular orbital (LUMO) and the highest occupied molecular orbital (HOMO) levels before the interaction of M68N with In-TCPP. FL: Fermi level. **b** Schematic of interface charge transfer in the M68N@In-TCPP heterostructure.

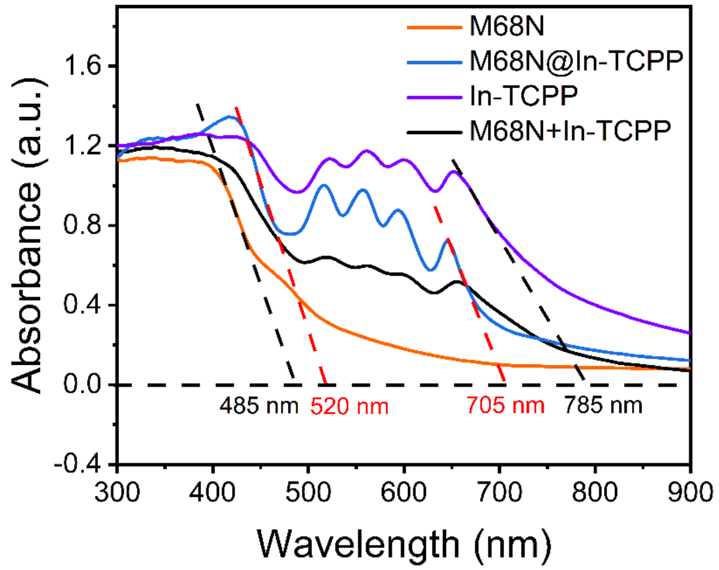

Supplementary Fig. 54. UV-visible diffuse reflectance spectra. Changes in the light absorption before and after the formation of M68N@In-TCPP heterostructure. Source data are provided as a Source Data file.

Pristine M68N has an absorption band edge identified at 485 nm, indicating that wavelengths longer than 485 nm are ineffective in exciting electrons from the HOMO to the LUMO in M68N. However, the heterostructure demonstrates a significant enhancement in absorption within the 400 nm to 650 nm wavelength range, alongside noticeable shifts in absorption peak wavelengths compared to the mechanical mixture. Concurrently, the Q-band absorption edge of In-TCPP, originally at 785 nm, exhibits a blue shift to 705 nm within the heterostructure. When compared to both the physical mixture of the two MOFs and the pure M68N, the heterostructure demonstrates a marked enhancement in visible light absorption, accompanied by perceptible shifts in the wavelengths of the absorption peaks. These phenomena suggest that the lattice oxygen vacancies at In-O bonds interface defects alter the band gaps and energy levels, facilitating longer wavelength irradiation to excite electrons from M68N's HOMO within the heterostructure. This interface formation allows for the excitation of more

electrons from M68N's HOMO to interface-associated energy levels under irradiation at wavelengths longer than 485 nm, compared to both the mechanical mixture and pure M68N. These excited electrons then transfer to the HOMO of In-TCPP across the heterojunctions and recombine with the holes within In-TCPP. This mechanism enables the excitation of electrons from the HOMO level of M68N under long wavelengths, which leave positively charged holes that oxidize H_2O to H_2O_2. Simultaneously, the excited electrons migrate to the LUMO of In-TCPP, facilitating the reduction of CO_2 to the HCOOH.

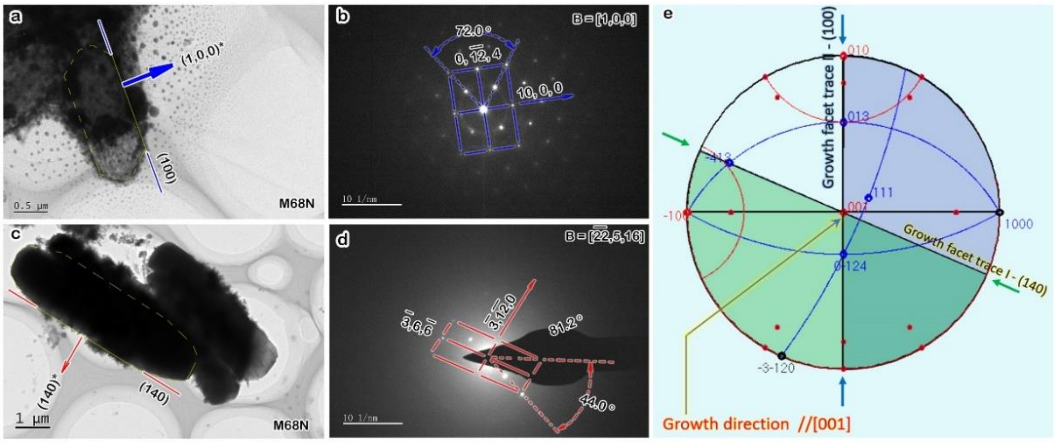

Supplementary Fig. 55. TEM. Phase identification and growth direction determination using TEM. **a** TEM Bright field image of an individual M68N nanorod with the annotation of one of the exposed facets (100) which is parallel to the growth direction. **b** The EDP obtained at [100] direction. **c** TEM bright field image of another M68N nanorod taken along [-22, 5, 16], around 2.3° away from [-413]. The exposed facet projected trace (140) is also parallel to the growth direction. **d** EDP obtained near zone axis direction [-22,5,16]. It is useful for indexing the projected facet (140). **e** A composited stereographic projection centered at [001]. The red spots denote orientation and the blue ones plane. The growth direction [001] is parallel to the common axis between the two parallel planes (100) and (140).

To further investigate the interfacial interaction and its structure, electronic diffraction pattern were taken under a low-Miller index zone axis perpendicular to the growth axis for the determination of crystallographic features of the growth morphology, *i e.*, growth direction, exposed facets, and 3D morphology. We observed many isolated M68N nanorods with double-tilting to obtain the electron diffraction pattern until the nanorod becomes electron irradiation damage. Supplementary Figs. 55a-c are transmission electron microscopy (TEM) bright field images of an M68N nanorod with the exposed facets and their projected facets parallel to the growth direction.

The electron diffraction pattern obtained from the two nanorods is shown in

Supplementary Figs. 55b-d which could be assigned to [100] and [-22, 5, 16] separately. The second diffraction pattern is a few degrees away from the zone axis due to the difficulty of systematically tilting the holder without causing the nanorod destroyed. The orthogonal lattice is not highly symmetric cubic and consequently, its planar Miller index is usually different from its planar normal except for special case. This makes electron diffraction pattern indexing not as convenient as the case for cubic structure. Since the growth direction is observed to fall into the planes (100) and (140), it could be deduced that the cross-product of the two planes is parallel to the growth direction and it reads [001]. This is schematically represented in the composited stereographic projection centered at [001] as shown in Supplementary Fig. 55e. The blue dots are planes and the red directions. It can be seen that the planar traces of (100) and (140) has a common crossing point [001].

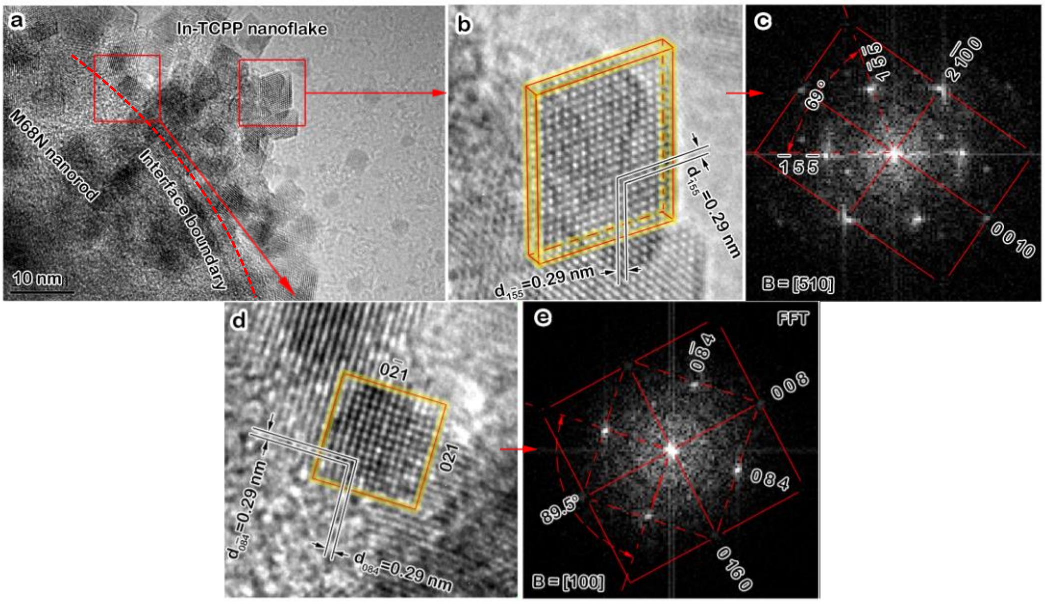

Supplementary Fig. 56. TEM. Determination of the morphology of In-TCPP mixed with M68N. **a** HRTEM image near the interface area, left side of the M68N nanorod, and the right side In-TCPP nanoflakes. The interface is not sharp due to large size of M68N. The two ROIs in red square are zoomed in and shown in **b** and **d**, which are transformed into FFT in **c** and **e**. The FFT images are indexed as the diffraction along [510] and [100].

To figure the interface out clearly, it is necessary to determine the growth morphology and the exposed facets of In-TCPP nanoflakes. Supplementary Fig. 56a shows a high-resolution TEM (HRTEM) image of an interface area of M68N and In-TCPP. It can be seen that the M68N nanorod is super large in size and thickness. It is not suitable for HRTEM imaging which requires a thickness less than 100 nm along the viewing direction (electron beam irradiation direction) at 200 KV. It is not strange the lattice fringe of M68N is not visible in this figure although it is still possible when imaging at the very thin top corner of the nanorod as shown in the in-set of Supplementary Fig. 56b. However, In-TCPP nanoflakes are very clear and take the same polygonal shape. By zooming the two ROIs in Supplementary Fig. 56a marked

with hollow red squares and presented in Supplementary Figs. 56b and 56d, the lattice fringes were allowed to turn into fast Fourier transformation (FFT) followed by diffraction indexing. In Supplementary Figs. 56c and 56e, the two FFT patterns are assigned to the zone axes [510] and [100] separately. The result is able to index the exposed facets of the nanoflake. They are (02 square shape along [100] but diamond shape along [510]. Fig. 4c is the 3D model of the projection of the nanoflake. When the viewing directions are moved from [100] to [510] on (001) plane by 43°, the projected pattern changes from square to diamond, and the exposed facets (021) and (0-21) projects into zero-thickness trace to a few nanometer band. According to the above SEM and TEM observation and careful crystallographic analysis, it is safe to claim that the heterostructure of M68N@In-TCPP composes with M68N nanorod enclosed by curved surface and the long axis is parallel to [001], and with In-TCPP nanoflake enclosed by a broad (001) plane and narrow (021) and (0-21) facets. At the surface of the M68N nanorod, In-TCPP nanoflake may assembly with the nanorod by using all the exposed facets, of course, broader is stronger. On the other hand, although M68N nanorod takes curved surface, it could be regarded as a polygonal nanorod enveloped by large quantity of tiny facets holding high-miller index including those low-Miller index. As mentioned above, we are not able to simultaneously image the lattice fringes of both M68N nanorod and the In-TCPP nanoflakes at the interface boundary. On the base of microscopic and crystallographic analysis of the crystal growth of the heterostructure, it is possible to reconstruct the interface configuration between M68N and In-TCPP (denoted by m and t separately). They are orthogonal lattice and the lattice parameters are very close to each other. This similarity paves the way to utilize the notable near coincidence site lattice concept to find the potential planar matching pairs[13]. Since both structures are body-centered orthogonal lattices, by setting up the following

lattice correspondence:

$$\begin{cases} X\text{: } [100]_{\text{M68N}} // [001]_{\text{In-TCPP}} \\ Y\text{: } [010]_{\text{M68N}} // [010]_{\text{In-TCPP}} \\ Z\text{: } [001]_{\text{M68N}} // [100]_{\text{In-TCPP}} \end{cases}$$

The orientation relationship could be described a 90° rotated orthogonal-to-orthogonal relationship. This is the so-called generalized Bain lattice correspondence. The principal strains in the Bain strain matrix are determined by the ratio of each vector pair. It reads

$$B = \begin{pmatrix} \eta_1 & 0 & 0 \\ 0 & \eta_2 & 0 \\ 0 & 0 & \eta_3 \end{pmatrix} = \begin{pmatrix} \frac{a_m}{c_t} & 0 & 0 \\ 0 & \frac{b_m}{b_t} & 0 \\ 0 & 0 & \frac{c_m}{a_t} \end{pmatrix} = \begin{pmatrix} 1.1549 & 0 & 0 \\ 0 & 0.8910 & 0 \\ 0 & 0 & 0.9827 \end{pmatrix} \quad (8)$$

The Bain strain requires a small angle rotation to minimize lattice distortion after the two crystals grow on each other. According to the invariant deformation element model (IDE model) for diffusional phase transformation, the shortest vector in M68N should keep non-rotated after the growth finishes[14]. Using this condition, it can be found that the final rotation angle required to minimize the lattice transformation strain is around 2.4 °. This is almost the same as the initial Bain lattice correspondence. Under this orientation relationship, by using stereographic projection tool, the plane in M68N matching (100) in In-TCPP is (100) at the interface and that matching (021) in In-TCPP is (110) in M68N. Therefore, the possible interface configurations are the following two cases. Case I: $(001)_{\text{M68N}}$ // $(100)_{\text{In-TCPP}}$; Case II: $(110)_{\text{M68N}}$ // $(021)_{\text{In-TCPP}}$ or $(1\text{-}10)_{\text{M68N}}$ // $(0\text{-}21)_{\text{In-TCPP}}$. The two cases can be demonstrated using 3D atomic model in Fig. 4d and 4e separately. The models show that the In-O octahedrons are shared by M68N and In-TCPP at the interface boundary for providing a stable and strong interface structure.

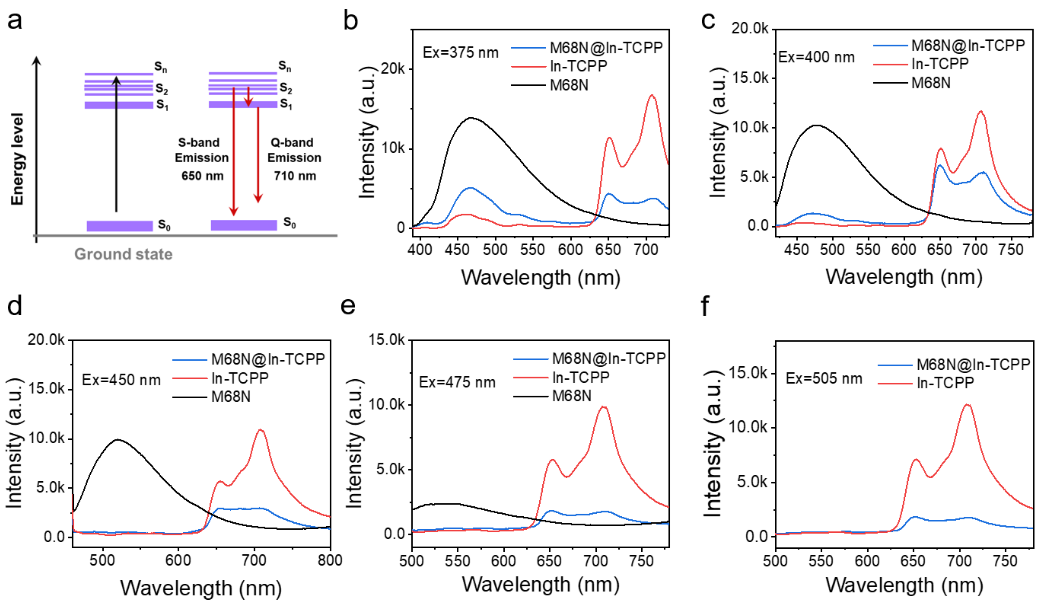

Supplementary Fig. 57. Photoluminescence spectra. **a** Schematic illustration of the Soret band and Q-band of the TCPP photoluminescence emission process. Photoluminescence (PL) spectra of M68N, M68N@In-TCPP, and In-TCPP at various excitation wavelengths **b** 375 nm, **c** 400 nm, **d** 450 nm, **e** 475 nm, and **f** 505 nm. Source data are provided as a Source Data file.

The photogenerated charge undergoes transfer from the ground state (S_0) to higher energy S_n levels, followed by relaxation to the levels of S_2 and S_1. Subsequent quenching events from S_2 to S_0 or S_1 to S_0 result in the emission of photoluminescence[15]. Specifically, the photoluminescence from S_2 to S_0 is attributed to the Soret-band (emission peaked at 650 nm, 1.91 eV), while the Q-band emission (peaked at 710 nm, 1.75 eV) arises from the transition of S_1 to S_0, with the former releasing higher energy. Notably, the intensity of the Q-band surpasses that of the S-band in In-TCPP, indicating a transfer of charge from S_2 to S_1, thereby enhancing the energy of the Q-band. This phenomenon is referred to as self-photoluminescence quenching. In the case of M68N@In-TCPP, a highly efficient charge transfer is observed in both the S-band and Q-band under five excitation wavelengths.

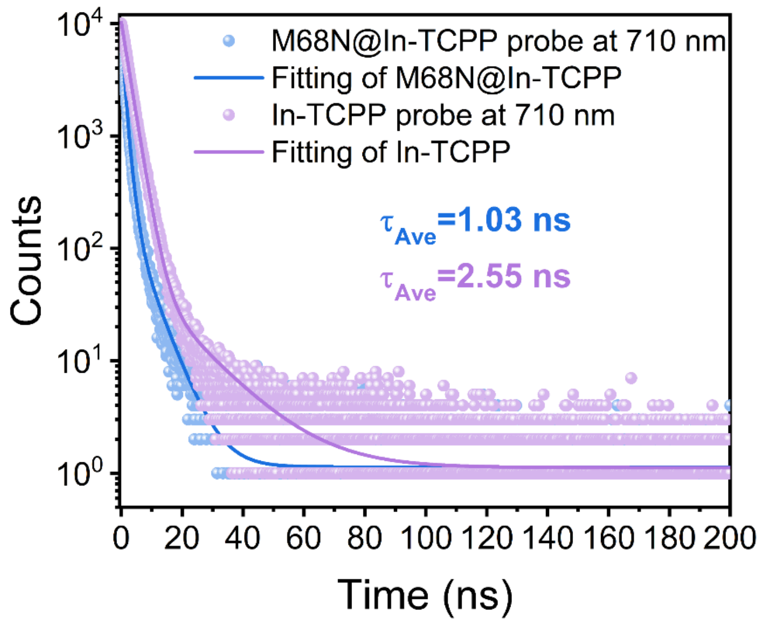

Supplementary Fig. 58. TRPL spectra. Time-resolved transient PL decay spectra of In-TCPP and M68N@In-TCPP. Excitation at 485 nm and emission probe delay at 710 nm. Source data are provided as a Source Data file.

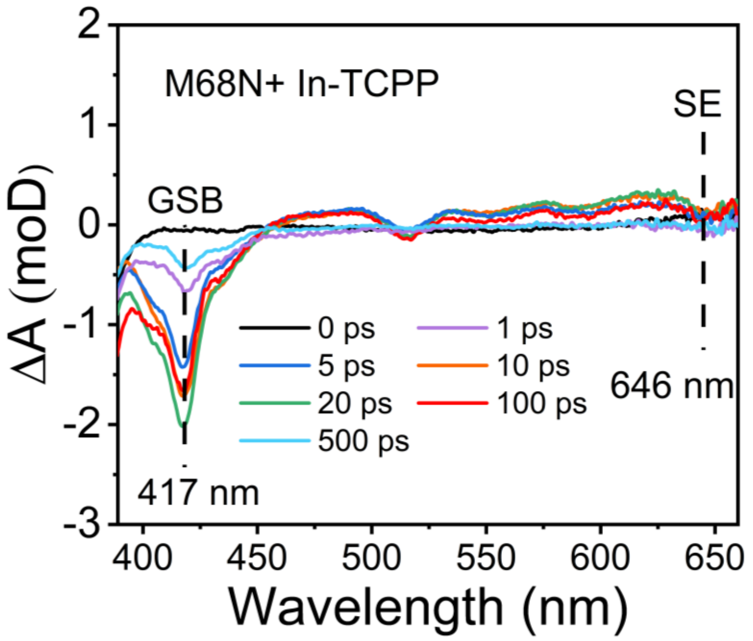

Supplementary Fig. 59. TA spectra. Ultrafast transient absorption spectra of M68N+In-TCPP: pump excitation at 380 nm. Source data are provided as a Source Data file.

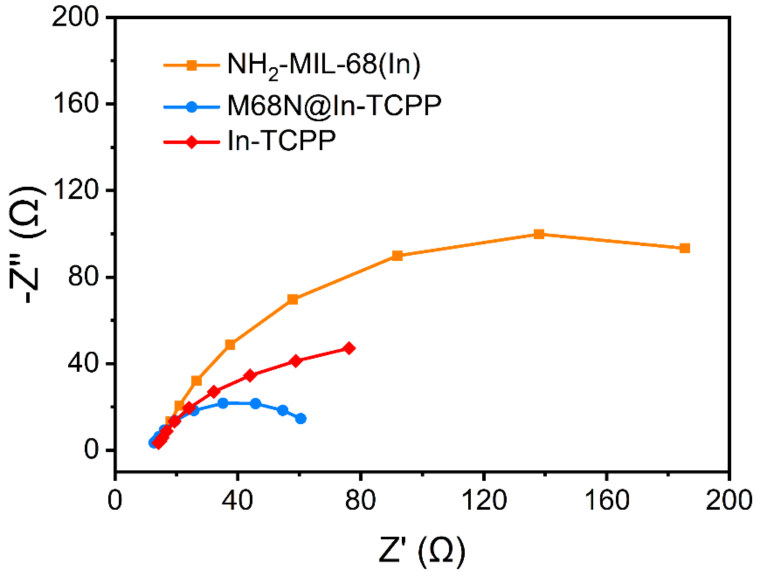

Supplementary Fig. 60. EIS. The electrochemical impedance spectroscopy (EIS) of NH_2-MIL-68(M68N), M68N@In-TCPP, and In-TCPP. Source data are provided as a Source Data file.

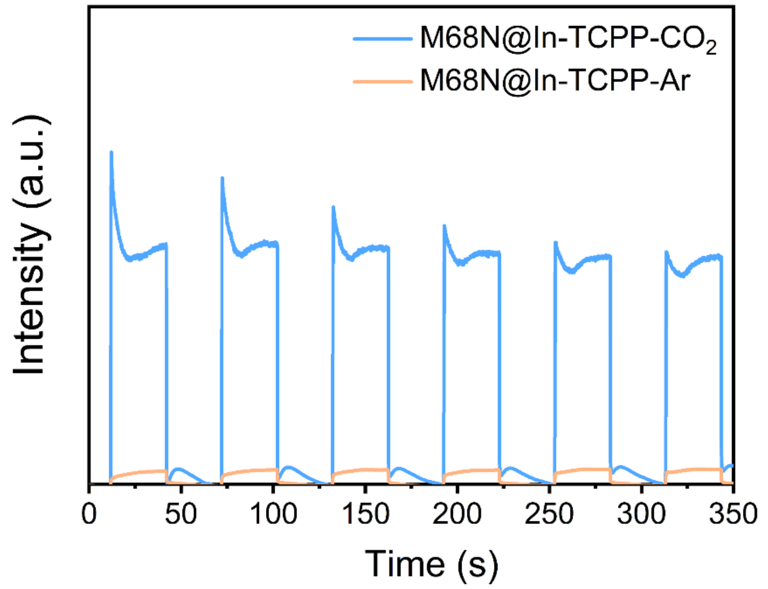

Supplementary Fig. 61. Photocurrent. The transient photocurrent of M68N@In-TCPP in Ar and CO_2 atmosphere. Source data are provided as a Source Data file.

Supplementary Tables

Supplementary Table 1. Performance comparison of known MOF/COF-based catalysts for overall photosynthesis and half CO_2 reduction reaction.

Photocatalyst	Light source	Reactants	Reduction product (μmol g^{-1} h^{-1})	Oxidation product (μmol g^{-1} h^{-1})	Products by adding SA and/or PS[a]	AQY (%)	Ref.
M68N@In-TCPP	300W Xe lamp (>400 nm)<br>Sunlight[b] (1.3 W cm^{-2})	CO_2, H_2O	HCOOH (121.1)<br>CO (22.3)<br>HCOOH (397.5)<br>CO (61.2)	H_2O_2 (119.3)<br>O_2 (5.9)<br>H_2O_2 (321.2)<br>O_2 (n.d.)	-	0.16 (420 nm)	This work
NH_2-UiO-66-TCPP	300W Xe lamp (>400 nm)	CO_2, H_2O	HCOOH (16.2)<br>CO (5.8)	O_2 (5.21)	-	-	[c]This work
CPM-5(In)/In-TCPP	300W Xe lamp (>400 nm)	CO_2, H_2O	HCOOH (22.1)<br>CO (1.8)	O_2 (4.86)	-	-	[d]This work
Fe-In-TCP (Porphyrin-based MOF)	300W Xe lamp (>400 nm)	CO_2, H_2O	HCOOH (17.6)	H_2O_2 (13.04)	-	n.r	[16]
MCOF-Ti_6Cu_3	300W Xe lamp (>400 nm)	CO_2, H_2O	HCOOH (169.0)	O_2 (n.r.[e])	-	n.r	[17]
PCN-222-Ni@UiO-67-NH_2	300W Xe lamp (>420 nm)	CO_2, H_2O	HCOOH (146)	H_2O_2 (n.r.)	-	n.r	[18]
NNU-31-Zn (Zn-based COF[f])	300W Xe lamp (>400 nm)	CO_2, H_2O	HCOOH (26.3)	O_2 (12.6)	-	0.035 (420 nm)	[19]
MAF-34-CoRu (Metal-azolate Framework)	300W Xe lamp (>400 nm)	CO_2, H_2O	CO (11.2)	O_2 (5.57)	-	0.015 (400 nm)	[20]
Bi-TTCOF-Zn	300W Xe lamp (>420 nm)	CO_2, H_2O	CO (11.56)	O_2 (5.8)	-	n.r	[21]
TTCOF-Zn (Zn-based Porphyrin COF)	300W Xe lamp (>400 nm)	CO_2, H_2O	CO (12.33)	O_2 (6.16)		0.00027 (420 nm)	[22]
TCOF-$MnMo_6$[g]	300W Xe lamp (>400 nm)	CO_2, H_2O	CO (37.3)	O_2 (18.65)	-	0.0067 (420 nm)	[23]
PET@NH_2-UiO-66	300W Xe lamp (>400 nm)	CO_2, H_2O	CO (6.7)<br>CH_4 (1.7)	H_2O_2 (n.r.)	-	n.r	[24]
$MAPbI_3$@PCN-221($Fe_{0.2}$)	300W Xe lamp (>400 nm)	CO_2, EA[i], H_2O	CO (4.16)<br>CH_4 (13)	O_2 (32.1)	-	n.r	[25]
Bi_3TiNbO_9-OVP[h]	300W Xe lamp (>400 nm)	$NaHCO_3$, H_2SO_4	CO (20.91)	O_2 (12.6)	-	0.46 (420 nm)	[26]
Vs-SnS_2	300W Xe lamp	CO_2, H_2O	CO (25.71)	O_2 (9)		0.028	[27]

	(Full light)					(420 nm)	
NH_2-Fe_3-Fe_2[j]	300W Xe lamp (>400 nm)	CO_2, MeCN/H_2O	n.r	n.r	HCOOH (395)	0.023 (365 nm)	28
66-IS-Ni[k]	300W Xe lamp (>400 nm)	CO_2, MeCN/H_2O	n.r	n.r	CO (1352)	0.14 (400 nm)	29
Fe-p-TMA	300W Xe lamp (>400 nm)	CO_2, MeCN/H_2O	n.r	n.r	CH_4 (1467)	0.18 (420 nm)	30
Eu-bpy-Ru-$CuCl_2$[l]	300W Xe lamp (>420 nm)	CO_2, MeCN/H_2O	n.d[m]	n.d	HCOOH (304)	n.r	31
(Co-based Porphyrin COF)	300W Xe lamp (>380 nm)	CO_2, MeCN/H_2O	n.r	n.r	HCOOH (93)	n.r	32
UiO67-Ir-Cou 6/Cu[n]	300W Xe lamp (>420 nm)	CO_2, MeCN/H_2O	n.d	n.d	$HCOO^-$ (408)	n.r	33
Ru(phen)$_3$-Eu-MOF	300W Xe lamp (>420 nm)	CO_2, MeCN/H_2O	n.d.	n.d	$HCOO^-$ (960)	n.r	34
MOF-808-EDTA	300W Xe lamp (>420 nm)	CO_2, MeCN/H_2O	n.d	n.d.	HCOOH (167)	n.r	35
pNJU-319Fe (Fe-porphyrin-based COFs)	300W Xe lamp (>420 nm)	CO_2, MeCN/H_2O	n.d	n.d.	CO (688) CH_4 (68.8)	n.r	36

Note: [a]SA: sacrificial agents; PS: photosensitizers;

[b]Sunlight: The sunlight intensity was gathered and enhanced by the condenser;

[c]NH_2-UiO-66 (Zr) with same NH_2-BDC ligand as M68N;

[d]In-carboxylate framework with same metal node as M68N (replace linker NH_2-BDC to 1,3,5-benzenetricarboxylate (BTC), CPM-5);

[e]n.r.: not reported;

[f]COF: covalent organic framework;

[g]TCOF-$MnMo_6$: confine amine-functionalized POM ($MnMo_6$-$2NH_2$) in TCOF.

[h]BNT-OVP: Bi_3TiNbO_9 nanosheets with oxygen vacancies;

[i]EA: ethylene glycol. [j]NH_2-Fe_3-Fe_2 = NH_2-H_2BDC = 2-aminobenzene-1,4-dicarboxylate acid;

[k]66-IS-Ni: NH_2-UiO-66/isatin-Schiff base Ni metal complexes;

[l]Eu-bpydc: bpydc = 2,2′-bipyridine-5,5′-dicarboxylate, integrate with Ru(phen)$_3$ photosensitizer derived tricarboxylate acid metalloligand;

[m]n.d.: not detect;

[n]UiO67-Ir-Cou 6: UiO-67 MOF integrate with Ir-bpy and coumarin 6 PS;

Supplementary Table 2. Inductively Coupled Plasma Mass Spectrometer (ICP-MS) analysis for M68N@In-TCPP.

Sample	In content (wt %)
M68N	27.67
In-TCPP(In-N-30%)	23.63
M68N@In-TCPP-(In-N-30%)[a]	27.20
M68N@In-TCPP-(In-N-47%)	27.55
M68N@In-TCPP-(In-N-60%)	27.79
M68N@In-TCPP-(In-N-75%)	28.05

Note: [a]: 30 % is the percentage of metallated TCPP(In) in In-TCPP.

Supplementary Table 3. Elemental Composition of the samples determined by XPS.

Samples	N atoms (%)	In atoms (%)	O atoms (%)	C atoms (%)	In/O ratio
M68N	1.74	7.97	42.36	47.93	0.19
M68N@In-TCPP-0.5 h	7.13	4.12	25.77	62.98	0.16
M68N@In-TCPP-1.0 h	5.76	4.88	27.78	61.58	0.18
M68N@In-TCPP-3.0 h	3.54	10.60	35.66	50.20	0.30
M68N@In-TCPP-5.0 h	3.27	13.55	35.45	47.73	0.38
In-TCPP	4.58	4.03	20.37	71.02	0.20

Supplementary Table 4. Comparison of solar to product conversion efficiency for CO_2 reduction in various reaction system.

Photocatalyst	Wavelength (nm)	Reactants	Sacrificial agents	Reduction product (μmol g^{-1} h^{-1})	Oxidation product (μmol g^{-1} h^{-1})	Solar to product conversion efficiency (*η* %)	Ref
[a]BNT	300 W Xe lamp (AM 1.5G)	$NaHCO_3$, H_2SO_4	-	CO (2.11)	O_2 (1.05)	0.0019	26
[b]BNT-OVP	300 W Xe lamp (AM 1.5G)	$NaHCO_3$, H_2SO_4	-	CO (20.91)	O_2 (12.6)	0.0214	26
$CuIn_5S_8$ single-unit-cell layers	300 W Xe lamp (AM 1.5G)	CO_2, H_2O	-	CH_4 (8.76)	O_2 (17.56)	0.017	37
methanosarcina barkeri-NiCu@CdS	75 W Xe lamp (AM 1.5G)	CO_2, sterilized autotrophic medium, 37 °C	Cysteine as sacrificial	CH_4 (79.38)	-	0.7	38
CotpyP-$SrTiO_3$:La,Rh|Au|RuO_2-$BiVO_4$	150 W solar light simulators (AM 1.5 G)	0.1 M $KHCO_3$ with CO_2, applied potential: +0.45 V vs RHE, current: 0.6 mA cm^{-2}	-	$HCOO^-$ (1.3 μmol h^{-1} cm^{-2})	O_2 (0.62 μmol h^{-1} cm^{-2})	0.08	39
S.ovata[c]/Cr_2O_3/Ru-$SrTiO_3$:La,Rh|ITO|RuO_2-$BiVO_4$: Mo.	150 W solar light simulators (AM 1.5 G)	N_2/CO_2 (80:20), *S. ovata* (CO_2-fixing acetogenic bacteria as medium), potential: +0.1 V vs SHE, current: 3 mA cm^{-2}		CH_3COO^- (9000)	O_2 (18000)	0.7	40
M68N@In-TCPP	Sunlight[d]	CO_2, H_2O	-	HCOOH (397.5)<br>CO (61.2)	H_2O_2 (321.2)<br>O_2 (not detected)	0.04	This work

Note: [a]BNT: Bi_3TiNbO_9 nanosheets; [b]BNT-OVP: Bi_3TiNbO_9 nanosheets with oxygen vacancies; [c]*S.ovata*： CO_2-fixing acetogenic bacteria. [d]Sunlight: The sunlight intensity was gathered and enhanced by the condenser.

Supplementary Table 5. Comparative experiments under different conditions for the artificial photosynthetic overall reaction of M68N@In-TCPP.

Reactant	Atmosphere	20 h of irradiation	Amount of the product after 20 h of reaction	
			Reduction product	Oxidation product
H_2O	CO_2	-	HCOOH (12.5 μmol)<br>CO (2.24 μmol)	H_2O_2 (12 μmol)<br>O_2(0.54 μmol)
H_2O	Ar	-	H_2 (1.98 μmol)	H_2O_2 (2.45 μmol)
[a]H_2O_2 (1.2 mM) in CH_3CN	Ar	H_2O_2 (1.16 mM)	[c]O_2 (n.d.)	H_2 (n.d.)
[b]HCOOH (1.3 mM) in CH_3CN	Ar	HCOOH (1.16 mM)	CO_2 (n.d.)	CO (0.53 μmol)<br>H_2 (n.d.)

Note: [a]adding H_2O_2 with the concentration equal to the H_2O oxidation yields over M68N@In-TCPP. [b]adding HCOOH with the concentration equal to the CO_2 reduction yields over M68N@In-TCPP. [c]n.d.: not detected.

Supplementary Table 6. ICP-MS analysis of the In content in the solution.

Amount of indium in the eluent	[a]M68N@In-TCPP
Before test	0
After 5 h of reaction	[b]n. d.
After 10 h of reaction	1.84 μg (0.13 wt%)
After 20 h of reaction	2.73 μg (0.19 wt %)

Note: [a]the content of In in M68N@In-TCPP is 27.55 wt%. [b]not detect.

Supplementary Table 7. The AQY of various photocatalysts at different Light wavelengths.

Light Wavelength (nm)	Samples			
	M68N	In-TCPP	M68N+In-TCPP	M68N@In-TCPP
365 nm (AQY %)	0.021	0.014	0.024	0.15
405 nm (AQY %)	0.020	0.014	0.024	0.16
420 nm (AQY %)	0.019	0.015	0.019	0.15
475 nm (AQY %)	0.006	0.014	0.014	0.10
505 nm (AQY %)	0.005	0.015	0.015	0.09
550 nm (AQY %)	-	0.011	0.011	0.07
600 nm (AQY %)	-	0.011	0.008	0.05
650 nm (AQY %)	-	0.011	0.009	0.04

Supplementary Table 8. The porosity and gas uptake of the samples.

Sample	S_{BET} [a] (m^2/g)	S_L [b] (m^2/g)	P. V. [c] (cm^3/g)	M. P. V. [d] (cm^3/g)	CO_2 uptake (cm^3/g or wt%)
M68N	675.9	1063.9	0.406	0.286	10.17 (2.03%)
M68N@InTCPP	798.4	1131.3	0.616	0.306	21.76 (3.98%)
In-TCPP	1107.3	1403.8	1.244	0.346	25.13 (4.93%)

Note: [a]Surface area calculated from N_2 adsorption isotherms at 77.3 *K* using the BET equation.

[b]Surface area calculated from N_2 adsorption isotherms at 77.3 *K* using the Langmuir equation.

[c]Pore volume (P. V.) calculated from nitrogen isotherm at P/P_0=0.995, 77.3 *K*.

[d]Micropore volume (M. P. V.) calculated from the nitrogen isotherm at P/P_0=0.050.

[e]CO^2 uptake was determined volumetrically using a Micromeritics ASAP 2020 M analyzer at 1.00 bar and 298.15 K

Supplementary Table 9. Assignment of in situ FT-IR bands observed on the surface of M68N and M68N@In-TCPP.

Samples	M68N peaks		M68N@In-TCPP		
	Wavenumber (cm^{-1})	Assignment	Wavenumber (cm^{-1})	Assignment	Reference
Dark adsorption	1454	HCO_3^-	1442	HCO_3^{--}	[41,42]
	1537	m-CO_3^{2-}	1524	m-CO_3^{2-}	[41,42]
	1650	CO_2^-	1637	CO_2^-	[41,42]
	1693	c-CO_3^{2-}	1690	c-CO_3^{2-}	[41,42]
Light Reaction	1330	H_2O_2	1320	H_2O_2	[42]
	1540, 1619	*COOH	1537, 1620	*COOH	[17,41,42]
	1742	HCOO*	1648, 1742	HCOO*	[17]

Supplementary Table 10. The fitting results of TRPL parameters for M68N@InTCPP and In-TCPP.

Samples	Excitation wavelength(nm)	Probe (nm)	Recovery times (ns)		Rel (%)		Average (ns)
M68N@InTCPP	375	475	τ_1	0.71	A_1	40.2	4.39
			τ_2	6.86	A_2	59.8	
		650	τ_1	0.75	A_1	53.7	1.61
			τ_2	2.60	A_2	46.4	
M68N	375	475	τ_1	0.36	A_1	71.2	0.78
			τ_2	1.85	A_2	28.8	
M68N@InTCPP	485	650	τ_1	1.27	A_1	29.8	0.71
			τ_2	0.48	A_2	70.2	
		710	τ_1	0.69	A_1	56.5	1.03
			τ_2	1.47	A_2	43.5	
In-TCPP	485	650	τ_1	1.41	A_1	72.9	1.81
			τ_2	2.89	A_2	27.1	
		710	τ_1	14.85	A_1	0.01	2.55
			τ_2	2.55	A_2	99.99	

Selective excitation of M68N at short wavelength of 375 nm presents the average lifetime (τ_{Ave}) of charge carriers in M68N as 0.78 ns. The formation of M68N@In-TCPP results in a much longer τ_{Ave} (4.39 ns), suggesting the improved efficiency of charge transfer process at the interface of M68N and In-TCPP. The laser with wavelength of 475 nm was chosen to induce the electron transition of In-TCPP and probe the emission of S- and Q- bands. The S-band emission of In-TCPP shows one exponential decay with lifetime of 1.81 ns, while the τ_{Ave} of M68N@In-TCPP is shortened to 0.71 ns. The Q-band emission of In-TCPP shows one-component decay profile with lifetime of 2.55 ns (τ_1 =14.85, 0.01%, τ_2 =2.55, 99.99%,). The decay with longer lifetime represents the photo-inducted charge transport, while the shorter one is

attributed to the porphyrin-localized exciton recombination. The extremely low percentage (0.01%) of decay with a longer lifetime (τ_1 =14.85) reflects the quick recombination of electron-hole in In-TCPP, whereas the Q-band emission of M68N@In-TCPP exhibits two decay components of τ_1 = 0.69 ns (56.5 %) and τ_2 = 1.47 ns (43.5%). Similarly, the percentage of long lifetime of M68N also increased from 28.8% to 59.8 % with the presence of M68N@In-TCPP heterostructure.

Supplementary Table 11. The fitting results of TA parameters for M68N@InTCPP and In-TCPP.

Samples	Probe (nm)	Recovery times (ps)		A		Per. (%)	Average (ps)
M68N@InTCPP	417	τ_1	7	A_1	0.0031	56.60	288
		τ_2	78	A_2	0.0012	24.52	
		τ_3	1402.5	A_3	0.0012	18.87	
	650	τ_1	9.3	A_1	$2.9*10^{-5}$	37.4	66
		τ_2	99.89	A_2	$4.9*10^{-5}$	62.6	
M68N+InTCPP	416	τ_1	308	A_1	0.427	42.7	
		τ_2	265	A_2	0.319	31.9	430
		τ_3	843	A_3	0.254	25.4	
	650	τ_1	503	A_1	0.717	71.7	449
		τ_2	303	A_2	0.293	29.3	
In-TCPP	412	τ_1	429.54	A_1	0.46	34.3	482
		τ_2	461.48	A_2	0.45	33.9	
		τ_3	561.2	A_3	0.42	31.8	
	643	τ_1	2.49	A_1	0.0012	79.1	494
		τ_2	2356.3	A_2	0.00033	20.9	

References

1. Perdew, J. P., Burke, K. & Ernzerhof, M. Generalized gradient approximation made simple. *Phys. Rev. Lett.* **77**, 3865-3868 (1996).
2. Kresse, G. & Furthmuller, J. Efficient iterative schemes for *ab initio* total-energy calculations using a plane-wave basis set. *Phys. Rev. B Condens. Matter.* **54**, 11169-11186 (1996).
3. Wu, L. *et al.* Amino-modified MIL-68(In) with enhanced hydrogen and carbon dioxide sorption enthalpy. *Microporous Mesoporous Mater.* **157**, 75-81 (2012).
4. Gao, Z. *et al.* Large π-conjugated indium-based metal-organic frameworks for high-performance electrochemical conversion of CO_2. *Nano Res.* **16**, 8743-8750 (2023).
5. Wang, Y. *et al.* Two-dimensional-on-three-dimensional metal-organic frameworks for photocatalytic H_2 production. *Angew. Chem. Int. Ed.* **61**, e202211031 (2022).
6. Zhao, Y., Wang, J. & Pei, R. Micron-sized ultrathin metal-organic frameworks sheet. *J. Am. Chem. Soc.* **142**, 10331-10336 (2020).
7. Wang, Z. *et al.* Efficient electroconversion of carbon dioxide to formate by a reconstructed amino-functionalized indium-organic framework electrocatalyst. *Angew. Chem. Int. Ed.* **60**, 19107-19112 (2021).
8. Zhang, Y. *et al.* H_2O_2 generation from O_2 and H_2O on a near-infrared absorbing porphyrin supramolecular photocatalyst. *Nat. Energy* **8**, 361-371 (2023).
9. Leng, F., Liu, H., Ding, M., Lin, Q.-P. & Jiang, H.-L. Boosting photocatalytic hydrogen production of porphyrinic MOFs: The metal location in metalloporphyrin matters. *ACS Catal.* **8**, 4583-4590 (2018).
10. Johnson, J. A., Zhang, X., Reeson, T. C., Chen, Y. S. & Zhang, J. Facile control of the charge density and photocatalytic activity of an anionic indium porphyrin framework via in situ metalation. *J. Am. Chem. Soc.* **136**, 15881-15884 (2014).
11. Sun, Y., Sun, L., Feng, D. & Zhou, H. C. An In situ one-pot synthetic approach towards multivariate zirconium MOFs. *Angew. Chem. Int. Ed.* **55**, 6471-6475 (2016).
12. Zheng, S.-T. *et al.* Pore Space Partition and Charge Separation in cage-within-cage indium−organic frameworks with high CO_2 uptake. *J. Am. Chem. Soc.* **132**, 17062-17064 (2010).
13. Sayle, D. C., Parker, S. C. & Harding, J. H. A study of thin film $YBa_2Cu_3O_{6.5}$/MgO interfaces using a near coincidence site lattice theory with atomistic simulation. *Mol. Simulat.* **12**, 127-139 (1994).
14. Liu, H., Waclawik, E. R. & Luo, C. A simplified invariant line analysis for face-centred cubic/body-centred cubic precipitation systems. Erratum. *J. Appl. Crystallogr.* **45**, 141-141 (2012).
15. Fang, Z. B. *et al.* Boosting interfacial charge-transfer kinetics for efficient overall CO_2 photoreduction via rational design of coordination spheres on metal-organic frameworks. *J. Am. Chem. Soc.* **142**, 12515-12523 (2020).

16. Chen, E. X. *et al.* Energy band alignment and redox-active sites in metalloporphyrin-spaced metal-catechol frameworks for enhanced CO_2 photoreduction. *Angew. Chem. Int. Ed.* **61**, e202111622 (2022).
17. Zhou, J. *et al.* Linking oxidative and reductive clusters to prepare crystalline porous catalysts for photocatalytic CO_2 reduction with H_2O. *Nat. Commun.* **13**, 4681 (2022).
18. Huang, H. B. *et al.* Engineering hierarchical architecture of metal-organic frameworks for highly efficient overall CO_2 photoreduction. *Small* **18**, e2200407 (2022).
19. Dong, L. Z. *et al.* Stable Heterometallic cluster-based organic framework catalysts for artificial photosynthesis. *Angew. Chem. Int. Ed.* **59**, 2659-2663 (2020).
20. Huang, N. Y. *et al.* Coupling ruthenium bipyridyl and cobalt imidazolate units in a metal-organic framework for an efficient photosynthetic overall reaction in diluted CO_2. *J. Am. Chem. Soc.* **144**, 8676-8682 (2022).
21. Li, Q. *et al.* Modulated connection modes of redox units in molecular junction covalent organic frameworks for artificial photosynthetic overall reaction. *J. Am. Chem. Soc.* **145**, 23167-23175 (2023).
22. Lu, M. *et al.* Rational Design of crystalline covalent organic frameworks for efficient CO_2 photoreduction with H_2O. *Angew. Chem. Int. Ed.* **58**, 12392-12397 (2019).
23. Lu, M. *et al.* Confining and highly dispersing single polyoxometalate clusters in covalent organic frameworks by covalent linkages for CO_2 photoreduction. *J. Am. Chem. Soc.* **144**, 1861-1871 (2022).
24. Li, P.-X. *et al.* Zirconium-based metal-organic frameworks particle films for visible-light-driven efficient photoreduction of CO_2. *ACS Sustain. Chem. & Eng.* **9**, 2319-2325 (2021).
25. Wu, L. Y. *et al.* Encapsulating perovskite quantum dots in iron-based metal-organic frameworks (MOFs) for efficient photocatalytic CO_2 reduction. *Angew. Chem. Int. Ed.* **58**, 9491-9495 (2019).
26. Yu, H. *et al.* Synergy of ferroelectric polarization and oxygen vacancy to promote CO_2 photoreduction. *Nat Commun.* **12**, 4594 (2021).
27. Yin, S.; Zhao, X.; Jiang, E.; Yan, Y.; Zhou, P.; Huo, P., Boosting water decomposition by sulfur vacancies for efficient CO_2 photoreduction. *Energy Environ. Sci.***,** ***15***, 1556-1562 (2022).
28. Wang, Y.-J. *et al.* Inter-clusters synergy in iron-organic frameworks for efficient CO_2 photoreduction. *Appl. Catal., B* **300,** 120487 (2022).
29. Dong, Y.-L., Liu, H.-R., Wang, S.-M., Guan, G.-W. & Yang, Q.-Y. Immobilizing isatin-schiff base complexes in NH_2-UiO-66 for highly photocatalytic CO_2 reduction. *ACS Catal.* **13**, 2547-2554. (2023).
30. Rao, H., Schmidt, L. C., Bonin, J. & Robert, M. Visible-light-driven methane formation from CO_2 with a molecular iron catalyst. *Nature* **548**, 74-77 (2017).
31. Zhuo, T. C. *et al.* H-bond-mediated selectivity control of formate versus CO during CO_2 photoreduction with two cooperative Cu/X sites. *J. Am. Chem. Soc.* **143**, 6114-6122 (2021)

32 Gong, Y. N. *et al.* Regulating photocatalysis by spin-state manipulation of cobalt in covalent organic frameworks. *J. Am. Chem. Soc.* **142**, 16723-16731 (2020).

33. Guo, S. *et al.* Switching excited state distribution of metal-organic frameworks for dramatically boosting photocatalysis. *Angew. Chem. Int. Ed.* **61**, e202206193 (2022).

34. Yan, Z. H. *et al.* Photo-generated dinuclear $Eu(II)_2$ active sites for selective CO_2 reduction in a photosensitizing metal-organic framework. *Nat. Commun.* **9**, 3353 (2018).

35. Li, J. *et al.* Self-adaptive dual-metal-site pairs in metal-organic frameworks for selective CO_2 photoreduction to CH_4. *Nat. Catal.* **4**, 719-729 (2021).

36. Dong, P. *et al.* Postsynthetic annulation of three-dimensional covalent organic frameworks for boosting CO_2 photoreduction. *J. Am. Chem. Soc.* **145**, 15473-15481 (2023).

37. Li, X. *et al.* Selective visible-light-driven photocatalytic CO_2 reduction to CH_4 mediated by atomically thin $CuIn_5S_8$ layers. *Nat. Energy* **4**, 690-699 (2019).

38. Ye, J. *et al.* Solar-driven methanogenesis with ultrahigh selectivity by turning down H_2 production at biotic-abiotic interface. *Nat. Commun.* **13**, 6612 (2022).

39. Wang, Q., Pornrungroj, C., Linley, S. & Reisner, E. Strategies to improve light utilization in solar fuel synthesis. *Nat. Energy* **7**, 13-24 (2021).

40. Wang, Q. *et al.* Molecularly engineered photocatalyst sheet for scalable solar formate production from carbon dioxide and water. *Nat. Energy* **5**, 703-710 (2020).

41. Gao, S. *et al.* Ingenious aartificial leaf based on covalent organic framework membranes for boosting CO_2 photoreduction. *J. Am. Chem. Soc.* **145**, 9520-9529 (2023).

42. Chen, X. *et al.* Bromo- and iodo-bridged building units in metal-organic frameworks for enhanced carrier transport and CO_2 photoreduction by water vapor. *Nat. Commun.* **13**, 4592 (2022).
