# Supplementary material for: Sunlight-driven simultaneous CO2 reduction and water oxidation using indium-organic framework heterostructures
Source: Nat Commun. 2025 Mar 16;16:2601. doi: 10.1038/s41467-025-57742-5 (PMC11911404; doi:10.1038/s41467-025-57742-5)
Supplement: Supplementary file 2 — Description of Additional Supplementary Files [file 41467_2025_57742_MOESM2_ESM.pdf]

Description of Additional supplementary file

Supplementary data 1:

Atomic coordinates of the optimized computational models
